# The word landscape of the non-coding segments of the *Arabidopsis thaliana *genome

**DOI:** 10.1186/1471-2164-10-463

**Published:** 2009-10-08

**Authors:** Jens Lichtenberg, Alper Yilmaz, Joshua D Welch, Kyle Kurz, Xiaoyu Liang, Frank Drews, Klaus Ecker, Stephen S Lee, Matt Geisler, Erich Grotewold, Lonnie R Welch

**Affiliations:** 1 Bioinformatics Laboratory, School of Electrical Engineering and Computer Science, Ohio University, Athens, Ohio, USA; 2 Department of Plant Cellular and Molecular Biology, Plant Biotechnology Center, The Ohio State University, Columbus, Ohio, USA; 3 Department of Statistics, University of Idaho, Moscow, Idaho, USA; 4 Department of Plant Biology, Southern Illinois University, Carbondale, Illinois, USA; 5 Biomedical Engineering Program, Ohio University, Athens, Ohio, USA; 6 Molecular and Cellular Biology Program, Ohio University, Athens, Ohio, USA

## Abstract

**Background:**

Genome sequences can be conceptualized as arrangements of motifs or words. The frequencies and positional distributions of these words within particular non-coding genomic segments provide important insights into how the words function in processes such as mRNA stability and regulation of gene expression.

**Results:**

Using an enumerative word discovery approach, we investigated the frequencies and positional distributions of all 65,536 different 8-letter words in the genome of *Arabidopsis thaliana*. Focusing on promoter regions, introns, and 3' and 5' untranslated regions (3'UTRs and 5'UTRs), we compared word frequencies in these segments to genome-wide frequencies. The statistically interesting words in each segment were clustered with similar words to generate motif logos. We investigated whether words were clustered at particular locations or were distributed randomly within each genomic segment, and we classified the words using gene expression information from public repositories. Finally, we investigated whether particular sets of words appeared together more frequently than others.

**Conclusion:**

Our studies provide a detailed view of the word composition of several segments of the non-coding portion of the *Arabidopsis *genome. Each segment contains a unique word-based signature. The respective signatures consist of the sets of enriched words, 'unwords', and word pairs within a segment, as well as the preferential locations and functional classifications for the signature words. Additionally, the positional distributions of enriched words within the segments highlight possible functional elements, and the co-associations of words in promoter regions likely represent the formation of higher order regulatory modules. This work is an important step toward fully cataloguing the functional elements of the *Arabidopsis *genome.

## Background

All genomes are composed of nucleotides, which are represented abstractly as letters (Adenine (A), Guanine (G), Cytosine (C), and Thymine (T)). Strings of such letters can be conceptualized as words, which provide the blueprints for organisms. Each word is found a specific number of times in a particular genome. Note that the expected frequency of a word is inversely related to the word's length. Some nucleotides appear more frequently than others (e.g. A/T in *Arabidopsis*), giving each genome a distinct (G+C)% content and biasing expected word frequencies. Higher order frequencies (dinucleotide and trinucleotide) also show distinct biases beyond those expected for single nucleotide frequencies [1].

Distinct selective pressures shape words positioned in different genomic regions. For example, a word in an open reading frame (ORF) has a direct influence on the primary amino acid sequence of a protein and hence is under strong selective pressure. In contrast, words in introns are likely to be under more relaxed selective constraints, unless they are important for gene functions, for example by providing docking sites for splicing factors [2] or for enzymes involved in the post-transcriptional processing of a transcript [3,4]. The gene sections corresponding to the 5' and 3' untranslated regions (5'UTRs and 3'UTRs, respectively) are also likely to be under less selective constraints than the ORFs, yet signatures of strong selection in UTRs have been described (reviewed in [5]). The constant formation of DNA microsatellites through slippage by the replication machinery, and the action of viruses and transposons, also complicate the word landscape, especially in regions with lower selective constraints (such as introns, UTRs and intergenic regions) [6,7].

This manuscript describes the results of a genome-wide analysis to discover putative regulatory words. Within this context, we define the *cis*-regulatory apparatus as all the DNA segments that are located proximal to a gene, and that also contribute to the gene's expression. It is the function of transcription factors, miRNAs, or other molecules that interact with DNA, to interpret the words (sequence code) hardwired in the *cis*-regulatory apparatus and to 'execute' them, thereby generating signals to the basal transcription machinery that result in changes to the rate of RNA production by the corresponding DNA-dependent RNA polymerases. When located upstream of the transcription start site (TSS), the *cis*-regulatory apparatus is often referred to as the promoter of a gene.

Promoters are typically divided into three regions: core, proximal and distal. The *core promoter*, a region at location [+1;-100] relative to the TSS, performs a central role in the formation of pre-initiation transcriptional complexes. Immediately upstream of the core promoter is the *proximal promoter*, which is located at position [-101;-1000] relative to the TSS and serves as a docking site for transcription factors. The *distal promoter *is located at [-1001;-3000] relative to the TSS and contains the regulatory elements that are commonly known as enhancers and silencers. The participation of a particular DNA segment in the regulation of gene expression can only be demonstrated experimentally. Thus, understanding the rules at play in deciphering the transcriptional regulatory code remains one of the most significant challenges in biology today.

Although most regulatory elements are present in the UTRs and upstream regions, due to their proximity to the TSS, studies have shown the presence of regulatory elements in introns, and, to a much lesser extent, in coding regions [2,8-16]. Building on this knowledge, a segment-based analysis was performed that is focused on non-coding regions within the open reading frames (i.e., introns) and flanking non-coding regions (i.e., UTRs and upstream regions). The coding regions were omitted from this analysis because they are under other selection pressures corresponding to the amino acid sequences of the proteins they produce, and thus they are subjected to biases other than regulation.

*Arabidopsis thaliana *provides an ideal reference organism to investigate the word landscape of a plant genome, and to relate said landscape to important biological features. The *Arabidopsis *genome consists of 125 Mbp arranged into five chromosomes [17,18]. The genome is well annotated and regions corresponding to introns, 3'UTRs, 5' UTRs, and intergenic genomic spaces are all available from The Arabidopsis Information Resource (TAIR, ) [19].

Many studies have characterized *Arabidopsis *DNA sequence motifs that participate in the regulation of particular genes (e.g., [20-23]), and public databases such as AthaMap [24] and AGRIS [25] provide comprehensive collections of *cis*-regulatory elements likely to participate in the regulation of gene expression. However, a systematic analysis of all the words present in the *Arabidopsis *genome is still lacking.

To analyze the different segments of the *Arabidopsis *genome, an enumerative word discovery approach was used to detect statistically overrepresented words. Similar approaches have been successfully applied over the last decade in the area of motif discovery [26-37]. In a 2005 study, Tompa et al. [38] showed that enumerative methods outperformed heuristic methods in many cases. They are particularly applicable in this research, because they allow the study of the entire 'word landscape' of a genomic data set.

Our approach scans the sequences and produces a set of words and word frequencies. This information is employed by a Markov model to compute expected word frequencies. Words with unexpectedly high frequencies are putative functional elements, and thus they are further characterized by comparing word frequencies and positions to gene induction or suppression using the method of Geisler et al. [39]. Additionally, clusters of similar words are formed and used to create motifs for putative transcription factor binding sites. Sequences that contain the same functional elements are grouped together into putative 'nodes' of regulatory networks. Words that co-occur often are identified as putative transcription factor binding modules.

## Results and Discussion

### Distribution of 8-letter words in the *Arabidopsis *genome

To determine the word distributions in the segments of the *Arabidopsis thaliana *genome that contribute to the *cis*-regulatory apparatus, a comprehensive analysis of 8-letter words in the entire genome was conducted and compared with segments corresponding to non-coding regions. Words of length 6-16 were examined and the complete results have been made available via AGRIS [25,40]. This article reports findings for words of length eight because they correspond to the typical DNA sequence length recognized by transcription factors (usually 6-8 bp [38,41]). Furthermore, 8-mers are long enough that there is enough diversity of word choices (~64,000) to reduce false positive results, while retaining sufficient word counts to be statistically informative.

The genome was sub-divided into segments comprising the 3' UTRs, 5'UTRs, promoters and introns (Table 1). The promoter segment was further dissected into the core promoter, corresponding to [-100; +1]; proximal promoter [-1000; -101]; and distal promoter [-3000; -1001]. The general properties of the six genome segments are shown in Table 1. As in a similar study, which was aimed at discovering regulatory elements involved in human DNA-repair pathways [26], word-based genomic signatures were created for each segment. Specifically, the following were identified for each of the genome segments: (1) the set of overrepresented words (signature words), (2) words missing from the sequences (*unwords*), (3) word-based clusters, (4) word co-occurrences and (5) functional categorizations of the signature words. The results are detailed in the remainder of this section.

**Table 1:** Segment characteristics for *Arabidopsis thaliana*

**Data Set**	**# Sequences/** **Chromosomes**	**Min. Seq. Length**	**Max. Seq. Length**	**Mean Seq. Length**	**Std. Deviation**	**Total Nucleotides**	**Genome Percentage**
3' UTRs	19,771	8	3,118	228.134	152.106	4,510,410	3.78

5' UTRs	18,585	8	3,214	140.088	130.288	2,603,531	2.18

Introns	118,319	8	10,234	164.446	178.484	19,457,029	16.32

Core Promoters	27,023	100	100	100	0	2,702,300	2.27

Proximal Promoters	27,023	900	900	900	0	24,320,700	20.41

Distal Promoters	27,025	1,371	2,000	1,999.96	5.01105	54,048,839	45.35

Genome-wide	5	18,585,000	30,432,600	23,837,300	4,432,780	119,186,497	100.00

Overview of the characteristics properties for non-coding segments and the entire genome for *Arabidopsis thaliana*. The number of sequence refers to the respective number of unique sequences in the specific segment. In case of the entire genome the sequences are the complete chromosomes. Min. Seq. Length refers to the length of the shortest sequence in the set, while Max. Seq. Length refers to the length of the longest sequence in the set. Mean Seq. Length provides the average length of the sequences in the set, while Std. Deviation describes the deviation from said mean. Finally Total Nucleotides describes the total number of nucleotides contained within the sequences of the set and Genome Percentage elaborates on the relationship between the nucleotide count of the set versus the entire genome.

Some sequences in the segments are shorter than 8 nucleotides. Since these sequences cannot harbour any putative regulatory elements in the context of this study, the sequences are removed from the table. For the 3'UTRs this results in a total of 179 nt being omitted, for 5'UTRs 1207 nt and for introns 26 nt. They are however included in the calculation of the background for the different segments since they contribute to the overall nucleotide distribution.

### Overrepresented Words

All 8-letter words present in the segments were identified and scored using *observed:expected *frequency ratios (*O/E*). Specifically, each word was scored and ranked by using the function S*ln(*S/E*_*S*_), where *S *is the number of sequences that contained the word, 'ln' is the natural logarithm, and *E*_*S *_is the number of sequences in which the word was expected to occur. Words discovered in the whole genome were analyzed using the O*ln(*O/E*_*O*_) score, with *O *referring to the overall occurrence of a word across the entire genome and *E*_*O *_representing the expected occurrence of that word. The 25 top-ranked words, corresponding to ~0.04% of all possible words, which also corresponds to ~0.04% of the discovered words, were taken as an exemplary subset of the results and further examined (see Table 2, 3, 4, 5, 6, 7, &8 and Additional file 1, 2, 3, 4, 5, 6, &7).

**Table 2:** The top 25 words in 3'UTRs

	**Unmasked**					**Masked**					**Unmasked**			
**Word**	**S**	**ES**	**O**	**EO**	**SlnSES**	**S**	**ES**	**O**	**EO**	**SlnSES**	**RevComp**	**RC_Pos**	**Pal**	**PValues**

TTTTTGTT	2264	2066.82	2488	2306.04	206.297	2279	2066.89	2501	2331.04	222.643	AACAAAAA	40	No	9.38E-05

TTTTTCTT	2171	1981.63	2404	2203.7	198.149	2183	1978.5	2427	2222.83	214.723	AAGAAAAA	49	No	1.34E-05

TTTTTTGG	998	824.458	1046	877.255	190.646	1003	831.208	1053	888.417	188.434	CCAAAAAA	651	No	1.71E-08

ATTTTGTA	732	583.938	752	615.741	165.421	738	599.956	759	634.768	152.831	TACAAAAT	37	No	6.00E-08

TAATTTTT	787	642.133	810	678.585	160.101	797	646.36	821	685.263	166.97	AAAAATTA	164	No	5.24E-07

ATGTTTTA	589	469.818	601	493.292	133.161	610	486.404	624	512.055	138.116	TAAAACAT	284	No	1.48E-06

TTTGTTTT	2517	2402.46	2847	2715.8	117.227	2555	2406.15	2897	2753.88	153.362	AAAACAAA	1963	No	0.006347

GTTTTTGA	491	390.189	504	408.466	112.838	512	407.532	527	427.529	116.841	TCAAAAAC	5031	No	2.76E-06

AAATTTTG	588	491.471	603	516.445	105.443	604	504.212	621	531.22	109.069	CAAAATTT	376	No	0.00011

ATTTTTTA	482	387.674	498	405.795	104.97	492	406.16	510	426.064	94.3317	TAAAAAAT	100	No	5.33E-06

ATTTTTCA	446	354.812	450	370.941	102.014	453	365.873	457	383.118	96.7633	TGAAAAAT	170	No	3.83E-05

TGTTTTGT	1227	1133.19	1326	1219.91	97.5897	1255	1162.02	1359	1260.07	96.6082	ACAAAACA	659	No	0.001413

ATAAAAAT	564	474.529	580	498.326	97.4203	566	480.088	581	505.265	93.1776	ATTTTTAT	27	No	0.000192

TTTTTTCT	1721	1628.11	1839	1786.09	95.4882	1722	1625.78	1847	1798.84	99.0176	AGAAAAAA	106	No	0.107802

AAAAATTG	397	312.488	400	326.178	95.0296	414	323.794	419	338.423	101.744	CAATTTTT	66	No	4.26E-05

TATAATAT	505	419.081	519	439.185	94.1802	514	429.108	530	450.594	92.7844	ATATTATA	275	No	0.000114

CTCTGTTT	763	674.497	814	713.654	94.0706	796	706.86	852	751.4	94.5386	AAACAGAG	227	No	0.000125

TTTTTAAT	897	808.297	929	859.536	93.4009	905	811.646	942	866.766	98.5274	ATTAAAAA	95	No	0.009964

TTCTTTTT	1884	1795.18	2075	1982.05	90.9811	1879	1764.9	2059	1964.59	117.709	AAAAAGAA	130	No	0.019465

TTTTTGGT	989	902.56	1029	963.191	90.453	1006	920.175	1052	987.344	89.7087	ACCAAAAA	9144	No	0.018455

ATTTTCTG	324	245.197	330	255.296	90.2932	340	264.756	346	275.991	85.047	CAGAAAAT	241	No	4.24E-06

AATATATT	462	382.795	474	400.615	86.8857	477	412.829	490	433.187	68.9186	AATATATT	21	Yes	0.000195

TTTGTGTG	688	607.303	705	640.94	85.8355	705	625.577	726	662.623	84.2635	CACACAAA	8153	No	0.006617

TGTTTTTT	1716	1632.37	1839	1791.05	85.7404	1730	1636.78	1864	1811.88	95.8269	AAAAAACA	1065	No	0.131261

Top 25 overrepresented words for the 3'Untranslated Regions in *Arabidopsis thaliana*. The *Word *attribute describes the short nucleotide sequence associated with a putative word. *S *and *ES *describe the number of sequences a word occurs in and the number of sequences the word was expected to occur in respectively, while *O *and *EO *describe the total number of occurrences and the expected total number of occurrences. The score *SlnSES *describes a statistical coverage of the sequences analyzed in the set and is based on a Markov Chain Background Model. Each set of attributes was computed for the masked as well as the unmasked version of the corresponding segment with the emphasis placed on the unmasked version (i.e. sorting of the table based on the unmasked *SlnSES *score).

Further information for the word is provided through its reverse complement (*RevComp*) and the position of the reverse complement in the set of results (*RC_Pos*) as well as a notion describing if the word is a genomic palindrome (*Pal*).

Finally, *PValues *describes a p-value that is assigned in order to provide statistical insight allowing the determination if a word is relevant or was discovered as interesting by random chance.

**Table 3:** The top 25 words in 5'UTRs

	**Unmasked**					**Masked**					**Unmasked**			
**Word**	**S**	**ES**	**O**	**EO**	**SlnSES**	**S**	**ES**	**O**	**EO**	**SlnSES**	**RevComp**	**RC_Pos**	**Pal**	**PValues**

CTCTTCTC	871	614.433	992	668.648	303.928	883	669.295	972	729.203	244.68	GAGAAGAG	4	No	-2.22E-16

CTTTCTCT	1154	1003.84	1293	1115.45	160.868	1204	1040.02	1327	1164.52	176.278	AGAGAAAG	15	No	1.14E-07

AACAAAAA	1051	920.535	1134	1018.31	139.302	1082	933.212	1157	1036.72	160.064	TTTTTGTT	16	No	0.000192

TTTCTTCA	611	492.734	631	532.75	131.443	808	714.439	849	780.981	99.4364	TGAAGAAA	227	No	1.88E-05

GAGAAGAG	316	211.511	360	225.309	126.863	305	219.262	327	231.047	100.664	CTCTTCTC	0	No	0

TTCTCTCC	455	346.314	464	371.543	124.193	504	412.082	517	440.518	101.482	GGAGAGAA	130	No	2.11E-06

CTTTCTTC	883	771.778	929	846.965	118.876	960	807.394	1006	888.66	166.197	GAAGAAAG	87	No	0.00285

CTCTCTTT	1229	1116.97	1351	1248.77	117.468	1284	1161.65	1410	1312.47	128.577	AAAGAGAG	9	No	0.002211

TTTCTCTC	1421	1308.64	1554	1478.35	117.051	1494	1385.35	1636	1591.45	112.808	GAGAGAAA	74	No	0.025997

AAAGAGAG	666	561.408	709	609.221	113.781	625	511.53	649	550.867	125.216	CTCTCTTT	7	No	4.30E-05

AGAAAAAA	1078	972.588	1154	1078.91	110.928	1097	983.999	1179	1097.24	119.255	TTTTTTCT	93	No	0.012195

AAAGAAAA	978	875.456	1093	966.097	108.328	1000	886.23	1111	981.116	120.779	TTTTCTTT	35	No	3.32E-05

ATCTCTCA	332	243.705	342	260.045	102.647	380	308.328	392	327.073	79.4223	TGAGAGAT	448	No	6.93E-07

AAAAAACA	759	663.266	803	723.672	102.333	774	675.404	814	736.19	105.466	TGTTTTTT	298	No	0.001952

TTTTTCTT	1020	923.944	1116	1022.27	100.884	1501	1398.57	1742	1608.22	106.097	AAGAAAAA	20	No	0.001995

AGAGAAAG	589	496.468	634	536.894	100.664	548	457.974	578	491.244	98.3457	CTTTCTCT	1	No	2.45E-05

TTTTTGTT	811	719.391	885	787.265	97.2085	1506	1441.03	1818	1662.31	66.4099	AACAAAAA	2	No	0.000332

ACAAAAAA	845	754.352	901	827.069	95.888	865	767.534	916	842.311	103.408	TTTTTTGT	37	No	0.005817

TAAAAAAG	231	152.899	238	162.371	95.3195	272	196.748	284	206.973	88.0952	CTTTTTTA	149	No	1.66E-08

CAAAAACC	357	273.395	362	292.183	95.2547	386	290.194	393	307.419	110.121	GGTTTTTG	59	No	4.45E-05

AAGAAAAA	1104	1013.1	1209	1126.3	94.8599	1134	1021.85	1230	1142.64	118.087	TTTTTCTT	14	No	0.007636

CCTCTCTT	351	268.225	358	286.579	94.4052	372	313.865	375	333.083	63.2147	AAGAGAGG	550	No	2.65E-05

TCTTCTCC	907	817.38	946	899.203	94.3624	899	804.147	934	884.875	100.239	GGAGAAGA	676	No	0.062179

TTCTCTCA	473	387.786	484	416.951	93.9572	538	481.457	555	517.331	59.7404	TGAGAGAA	126	No	0.000721

Top 25 overrepresented words for the 5'Untranslated Regions in *Arabidopsis thaliana*. The *Word *attribute describes the short nucleotide sequence associated with a putative word. *S *and *ES *describe the number of sequences a word occurs in and the number of sequences the word was expected to occur in respectively, while *O *and *EO *describe the total number of occurrences and the expected total number of occurrences. The score *SlnSES *describes a statistical coverage of the sequences analyzed in the set and is based on a Markov Chain Background Model. Each set of attributes was computed for the masked as well as the unmasked version of the corresponding segment with the emphasis placed on the unmasked version (i.e. sorting of the table based on the unmasked *SlnSES *score).

Further information for the word is provided through its reverse complement (*RevComp*) and the position of the reverse complement in the set of results (*RC_Pos*) as well as a notion describing if the word is a genomic palindrome (*Pal*).

Finally, *PValues *describes a p-value that is assigned in order to provide statistical insight allowing the determination if a word is relevant or was discovered as interesting by random chance.

**Table 4:** The top 25 words in Introns

	**Unmasked**					**Masked**					**Unmasked**			
**Word**	**S**	**ES**	**O**	**EO**	**SlnSES**	**S**	**ES**	**O**	**EO**	**SlnSES**	**RevComp**	**RC_Pos**	**Pal**	**PValues**

TTTTTGTT	10048	9365.74	11094	10679.8	706.524	9819	9103.26	10783	10355.3	743.17	TTTTTGTT	10048	9365.74	3.44E-05

TTTTTCTT	9144	8495.68	10021	9609.91	672.454	8939	8293.57	9751	9363.74	669.915	TTTTTCTT	9144	8495.68	1.58E-05

CTTTTTTC	2764	2170.42	2821	2314.32	668.224	2713	2187.97	2767	2333.43	583.515	CTTTTTTC	2764	2170.42	8.88E-16

GTTTTTGA	2673	2105.13	2742	2243.33	638.372	2631	2056.65	2696	2190.66	647.973	GTTTTTGA	2673	2105.13	-2.22E-16

TTTTGCAG	3505	2959.4	3523	3179.19	593.06	3452	2920.63	3470	3136.4	577.016	TTTTGCAG	3505	2959.4	1.07E-09

TTTTTTGT	7618	7067.97	8198	7889.79	570.901	7400	6823.86	7922	7600.06	599.8	TTTTTTGT	7618	7067.97	0.000286

TTTTTTGG	3765	3238.3	3942	3487.94	567.378	3635	3124.76	3795	3362.05	549.804	TTTTTTGG	3765	3238.3	2.62E-14

TTTTCTTT	9256	8733.23	10299	9900.39	538.109	9041	8500.1	9994	9615.3	557.761	TTTTCTTT	9256	8733.23	3.48E-05

TGTTTTTT	7487	6984.58	8028	7790.67	520.072	7254	6759.65	7750	7524.05	512	TGTTTTTT	7487	6984.58	0.003768

CTCTCTTT	3193	2716.79	3289	2911.9	515.697	3086	2625.01	3165	2811.09	499.291	CTCTCTTT	3193	2716.79	3.97E-12

ATTTTTTA	2508	2044.78	2645	2177.76	512.128	2383	2003.78	2486	2133.28	413.027	ATTTTTTA	2508	2044.78	3.33E-16

TTTTTTCC	3166	2702.47	3253	2896.16	501.186	3086	2616.31	3161	2801.55	509.528	TTTTTTCC	3166	2702.47	4.13E-11

TGTTTCAG	2215	1790.21	2239	1902.05	471.614	2153	1745.3	2177	1853.55	451.987	TGTTTCAG	2215	1790.21	3.01E-14

GGTTTTTG	2029	1611.17	2092	1708.92	467.851	1997	1584.97	2058	1680.71	461.47	GGTTTTTG	2029	1611.17	1.11E-16

TTTTGTTT	12142	11689.3	13879	13619.2	461.327	11843	11368.1	13438	13205.7	484.659	TTTTGTTT	12142	11689.3	0.013306

TTTGTTTT	11017	10569.9	12527	12188.1	456.39	10729	10259.7	12106	11796.5	479.827	TTTGTTTT	11017	10569.9	0.00113

CTTTTTTA	2234	1828.76	2282	1943.72	447.149	2178	1816.31	2220	1930.26	395.524	CTTTTTTA	2234	1828.76	4.17E-14

AATATATT	2022	1642.55	2143	1742.72	420.253	1925	1679.14	2019	1782.16	263.038	AATATATT	2022	1642.55	4.44E-16

ATTTTTCA	2411	2030.35	2467	2162.1	414.291	2349	1971.89	2398	2098.68	411.073	ATTTTTCA	2411	2030.35	7.51E-11

ATTTTTTC	2810	2425.9	2881	2592.99	413.021	2736	2412.96	2800	2578.85	343.758	ATTTTTTC	2810	2425.9	1.43E-08

CAATTTTT	2402	2023.84	2481	2155.04	411.472	2320	1952.98	2388	2078.19	399.534	CAATTTTT	2402	2023.84	3.73E-12

TTTTTTCT	7674	7280.17	8254	8142.69	404.295	7476	7074.7	8001	7897.8	412.475	TTTTTTCT	7674	7280.17	0.109849

TGTTGCAG	1922	1563.72	1933	1657.84	396.507	1891	1543.21	1902	1635.78	384.332	TGTTGCAG	1922	1563.72	2.42E-11

TTTCATTT	4636	4258.39	4840	4630.74	393.879	4538	4169.05	4731	4529.8	384.813	TTTCATTT	4636	4258.39	0.001152

TTTTTATT	5647	5276.08	6142	5792.21	383.658	5417	5037.47	5842	5517.96	393.481	TTTTTATT	5647	5276.08	2.72E-06

Top 25 overrepresented words for the Introns in *Arabidopsis thaliana*. The *Word *attribute describes the short nucleotide sequence associated with a putative word. *S *and *ES *describe the number of sequences a word occurs in and the number of sequences the word was expected to occur in respectively, while *O *and *EO *describe the total number of occurrences and the expected total number of occurrences. The score *SlnSES *describes a statistical coverage of the sequences analyzed in the set and is based on a Markov Chain Background Model. Each set of attributes was computed for the masked as well as the unmasked version of the corresponding segment with the emphasis placed on the unmasked version (i.e. sorting of the table based on the unmasked *SlnSES *score).

Further information for the word is provided through its reverse complement (*RevComp*) and the position of the reverse complement in the set of results (*RC_Pos*) as well as a notion describing if the word is a genomic palindrome (*Pal*).

Finally, *PValues *describes a p-value that is assigned in order to provide statistical insight allowing the determination if a word is relevant or was discovered as interesting by random chance.

**Table 5:** The top 25 words in Core Promoters

	**Unmasked**					**Masked**					**Unmasked**			
**Word**	**S**	**ES**	**O**	**EO**	**SlnSES**	**S**	**ES**	**O**	**EO**	**SlnSES**	**RevComp**	**RC_Pos**	**Pal**	**PValues**

TATAAATA	1355	1071.69	1369	1175.57	317.831	1300	1029.92	1311	1128.85	302.753	TATTTATA	69	No	2.02E-08

CTATAAAT	712	474.27	716	514.446	289.286	704	464.711	708	503.987	292.416	ATTTATAG	2504	No	7.77E-16

CTATATAA	636	410.261	638	444.486	278.826	626	450.579	628	488.533	205.839	TTATATAG	18530	No	1.11E-16

ATATAAAC	560	350.797	560	379.643	261.928	554	347.685	554	376.253	258.091	GTTTATAT	26957	No	4.44E-16

TAAAAAAT	473	295.342	480	319.301	222.765	453	298.58	460	322.82	188.835	ATTTTTTA	12	No	-2.22E-16

ATATATAC	544	394.869	559	427.688	174.295	507	330.093	515	357.099	217.573	GTATATAT	5651	No	7.41E-10

AATATATT	300	181.346	300	195.646	151.012	287	195.452	287	210.918	110.256	AATATATT	6	Yes	2.74E-12

TTATATAA	524	397.031	529	430.047	145.398	514	430.79	518	466.905	90.7739	TTATATAA	7	Yes	2.22E-06

AAGAAAAA	1261	1129.24	1318	1240.05	139.165	1189	1063	1238	1165.84	133.189	TTTTTCTT	25	No	0.014544

ATATAAAG	378	262.861	380	284.014	137.316	375	261.181	377	282.19	135.643	CTTTATAT	377	No	3.41E-08

TATATAAA	1260	1131.11	1276	1242.15	135.966	1234	1102.41	1250	1209.97	139.143	TTTATATA	1458	No	0.171817

AGAAAAAA	1127	1000.04	1170	1095.49	134.693	1063	936.863	1099	1025.06	134.271	TTTTTTCT	31	No	0.01331

ATTTTTTA	312	204.097	315	220.282	132.415	299	207.163	302	223.604	109.715	TAAAAAAT	4	No	1.17E-09

TTTTAAAA	688	568.245	696	617.46	131.571	658	543.865	665	590.7	125.351	TTTTAAAA	13	Yes	0.001019

CTCTTCTC	402	294.202	429	318.061	125.499	371	277.661	390	300.087	107.516	GAGAAGAG	444	No	1.97E-09

ACAAAAAA	958	840.585	988	918.052	125.259	917	799.552	939	872.564	125.681	TTTTTTGT	45	No	0.011607

ATAAATAC	578	466.039	582	505.44	124.446	574	459.992	578	498.825	127.095	GTATTTAT	14072	No	0.000465

TTATAAAA	507	397.553	508	430.617	123.294	490	386.47	491	418.525	116.302	TTTTATAA	945	No	0.000153

AAATTAAA	718	609.913	745	663.251	117.144	682	578.03	705	628.206	112.806	TTTAATTT	96	No	0.000967

GCCCATTA	374	273.89	396	295.991	116.512	372	272.658	394	294.653	115.571	TAATGGGC	190	No	1.82E-08

AAAAAACA	893	787.368	924	859.073	112.42	849	736.927	874	803.277	120.193	TGTTTTTT	33	No	0.014723

TTAAAAAA	805	701.565	828	764.227	110.71	768	667.112	788	726.227	108.159	TTTTTTAA	27	No	0.01177

ATTAAAAA	708	609.58	719	662.885	105.969	671	581.412	681	631.921	96.1611	TTTTTAAT	316	No	0.016276

GCCCAATA	322	231.782	340	250.291	105.859	321	228.286	337	246.5	109.41	TATTGGGC	130	No	4.26E-08

Top 25 overrepresented words for the core promoter regions in *Arabidopsis thaliana*. The *Word *attribute describes the short nucleotide sequence associated with a putative word. *S *and *ES *describe the number of sequences a word occurs in and the number of sequences the word was expected to occur in respectively, while *O *and *EO *describe the total number of occurrences and the expected total number of occurrences. The score *SlnSES *describes a statistical coverage of the sequences analyzed in the set and is based on a Markov Chain Background Model. Each set of attributes was computed for the masked as well as the unmasked version of the corresponding segment with the emphasis placed on the unmasked version (i.e. sorting of the table based on the unmasked *SlnSES *score).

Further information for the word is provided through its reverse complement (*RevComp*) and the position of the reverse complement in the set of results (*RC_Pos*) as well as a notion describing if the word is a genomic palindrome (*Pal*).

Finally, *PValues *describes a p-value that is assigned in order to provide statistical insight allowing the determination if a word is relevant or was discovered as interesting by random chance.

**Table 6:** The top 25 words in Proximal Promoters

	**Unmasked**					**Masked**					**Unmasked**			
**Word**	**S**	**ES**	**O**	**EO**	**SlnSES**	**S**	**ES**	**O**	**EO**	**SlnSES**	**RevComp**	**RC_Pos**	**Pal**	**PValues**

TAAAAAAT	4249	3411.11	4837	3674.74	933.272	3681	3028.65	4071	3237.18	718.039	ATTTTTTA	1	No	0

ATTTTTTA	3876	3135.31	4372	3358.5	822.011	3313	2758.58	3636	2932.38	606.738	TAAAAAAT	0	No	2.22E-16

TTATATAA	3094	2505.92	3390	2650.31	652.239	2712	2508.38	2934	2653.02	211.674	TTATATAA	2	Yes	7.77E-16

AATATATT	3636	3104.08	4093	3322.92	575.097	3178	3009.54	3503	3215.49	173.09	AATATATT	3	Yes	1.67E-15

GAAAAAAG	2066	1652.5	2182	1718.49	461.395	1956	1621.19	2053	1684.9	367.226	CTTTTTTC	5	No	1.11E-16

CTTTTTTC	1960	1578.31	2072	1638.97	424.512	1869	1559.58	1969	1618.92	338.269	GAAAAAAG	4	No	1.11E-16

AAAAATTG	2975	2595.17	3208	2749.61	406.363	2737	2368.41	2938	2497.98	395.888	CAATTTTT	9	No	-6.66E-16

TAAAATTT	4339	3951.48	5058	4305.15	405.93	3764	3348.9	4214	3603.07	439.821	AAATTTTA	10	No	-6.66E-16

TAATTTTT	4656	4272.02	5336	4686.12	400.739	4125	3726.41	4609	4040.78	419.188	AAAAATTA	19	No	0

CAATTTTT	2872	2499.79	3110	2643.5	398.638	2633	2269.83	2829	2389.32	390.785	AAAAATTG	6	No	6.66E-16

AAATTTTA	4239	3880.57	4921	4221.59	374.5	3651	3305.77	4102	3553.5	362.665	TAAAATTT	7	No	8.88E-16

TACAAAAT	2589	2241.1	2821	2357.73	373.61	2344	2040.96	2514	2138.69	324.496	ATTTTGTA	26	No	6.66E-16

ATTTTCTA	2206	1886.09	2346	1970.39	345.622	2022	1748.93	2142	1822.19	293.357	TAGAAAAT	17	No	8.88E-16

TGAAAAAT	2374	2075.6	2517	2176.47	318.891	2230	1927.32	2354	2015.09	325.288	ATTTTTCA	21	No	5.64E-13

AAAAAATC	3874	3607.85	4265	3902.57	275.738	3494	3280.06	3823	3524	220.77	GATTTTTT	68	No	5.63E-09

CATTTTTC	1675	1426.93	1760	1477.44	268.478	1558	1356.8	1624	1402.92	215.428	GAAAAATG	29	No	5.16E-13

TAAGAAAT	1895	1645.36	1990	1710.83	267.683	1773	1553.49	1856	1612.42	234.336	ATTTCTTA	23	No	2.52E-11

TAGAAAAT	2154	1904.65	2281	1990.5	265.005	1971	1754.61	2083	1828.31	229.215	ATTTTCTA	12	No	1.04E-10

GGAAAAAA	2679	2426.86	2853	2562.63	264.801	2506	2238.07	2643	2354.4	283.363	TTTTTTCC	98	No	9.20E-09

AAAAATTA	4735	4477.84	5547	4933.58	264.404	4109	3862.67	4667	4200.51	254.025	TAATTTTT	8	No	1.33E-15

CAAAATTT	3347	3092.9	3655	3310.2	264.267	3054	2796.42	3304	2974.88	269.093	AAATTTTG	60	No	1.95E-09

ATTTTTCA	2338	2088.5	2489	2190.56	263.846	2169	1928.62	2295	2016.5	254.769	TGAAAAAT	13	No	2.29E-10

TTTTTTGG	3369	3120.79	3724	3341.96	257.829	3050	2802.67	3330	2981.91	257.935	CCAAAAAA	28	No	4.49E-11

ATTTCTTA	1947	1705.79	2052	1775.75	257.518	1800	1598.57	1900	1660.66	213.623	TAAGAAAT	16	No	8.37E-11

Top 25 overrepresented words for the proximal promoters in *Arabidopsis thaliana*. The *Word *attribute describes the short nucleotide sequence associated with a putative word. *S *and *ES *describe the number of sequences a word occurs in and the number of sequences the word was expected to occur in respectively, while *O *and *EO *describe the total number of occurrences and the expected total number of occurrences. The score *SlnSES *describes a statistical coverage of the sequences analyzed in the set and is based on a Markov Chain Background Model. Each set of attributes was computed for the masked as well as the unmasked version of the corresponding segment with the emphasis placed on the unmasked version (i.e. sorting of the table based on the unmasked *SlnSES *score).

Further information for the word is provided through its reverse complement (*RevComp*) and the position of the reverse complement in the set of results (*RC_Pos*) as well as a notion describing if the word is a genomic palindrome (*Pal*).

Finally, *PValues *describes a p-value that is assigned in order to provide statistical insight allowing the determination if a word is relevant or was discovered as interesting by random chance.

**Table 7:** The top 25 words in Distal Promoters

	**Unmasked**					**Masked**					**Unmasked**			
**Word**	**S**	**ES**	**O**	**EO**	**SlnSES**	**S**	**ES**	**O**	**EO**	**SlnSES**	**RevComp**	**RC_Pos**	**Pal**	**PValues**

ATTTTTTA	5789	4874.02	7202	5393.37	995.937	4920	4189.9	5773	4568.53	790.309	TAAAAAAT	1	No	6.66E-16

TAAAAAAT	5865	4983.57	7314	5527.8	955.154	5003	4269.17	5877	4662.83	793.568	ATTTTTTA	0	No	6.66E-16

GAAAAAAG	3578	2825.77	3921	2995.09	844.484	3394	2744.34	3697	2903.99	721.112	CTTTTTTC	3	No	8.88E-16

CTTTTTTC	3546	2878.92	3904	3054.71	739.005	3345	2798.31	3662	2964.33	596.918	GAAAAAAG	2	No	0

TTATATAA	4781	4107.17	5656	4470.46	726.305	4138	3955.09	4717	4291.1	187.08	TTATATAA	4	Yes	0

AATATATT	5432	4895.21	6702	5419.31	565.205	4688	4574.65	5538	5029.33	114.742	AATATATT	5	Yes	0

CAAGAAAC	2910	2459.44	3187	2587.64	489.513	2818	2410.32	3089	2533.47	440.364	GTTTCTTG	7	No	-4.44E-16

GTTTCTTG	2912	2482.93	3182	2613.58	464.176	2842	2430.36	3108	2555.55	444.685	CAAGAAAC	6	No	0

GAAAAATG	3158	2736.51	3416	2895.24	452.402	2871	2566.09	3080	2705.63	322.343	CATTTTTC	29	No	0

GTTTTTGA	3516	3093.27	3830	3296.52	450.382	3207	2816.69	3462	2984.91	416.186	TCAAAAAC	13	No	8.88E-16

GAAAAAAC	3013	2605.34	3240	2749.19	438.004	2744	2495.22	2935	2627.17	260.786	GTTTTTTC	26	No	5.55E-16

CAATTTTT	4457	4041.77	4991	4393.18	435.864	4009	3601.54	4440	3878.67	429.685	AAAAATTG	25	No	1.67E-15

ATTTTGTA	4098	3689.96	4626	3981.23	429.814	3735	3342.23	4123	3580.11	414.995	TACAAAAT	69	No	1.55E-15

TCAAAAAC	3414	3011.29	3688	3203.78	428.513	3129	2749.95	3358	2910.25	404.054	GTTTTTGA	9	No	7.77E-16

GAAGAAAG	3851	3448.5	4291	3702.07	425.126	3664	3290.44	4048	3520.87	394.006	CTTTCTTC	59	No	1.11E-16

GTTTTATG	2173	1793.07	2293	1861.81	417.607	2048	1720.91	2156	1784.36	356.372	CATAAAAC	57	No	1.11E-16

CTTTATTC	1618	1250.45	1676	1284.79	416.937	1500	1215.7	1548	1248.25	315.217	GAATAAAG	43	No	4.44E-16

GTTTTAAG	1957	1584.64	2054	1638.71	413.031	1791	1482.73	1871	1530.29	338.304	CTTAAAAC	28	No	1.33E-15

ATTTTTCA	4081	3695.36	4496	3987.5	405.1	3743	3364	4095	3605.05	399.585	TGAAAAAT	40	No	6.66E-16

TAAGAAGT	1465	1112.41	1517	1139.93	403.359	1388	1100.56	1435	1127.54	322.073	ACTTCTTA	62	No	-8.88E-16

CTTGTTTC	2351	1980.52	2504	2064.03	403.153	2269	1929.76	2415	2009.12	367.453	GAAACAAG	35	No	0

CAAAAAAG	3391	3011.99	3696	3204.57	401.915	3126	2864.52	3392	3038.54	273.068	CTTTTTTG	88	No	0

TAGAAAAT	3556	3178.38	3887	3393.13	399.217	3219	2901.76	3488	3080.38	333.981	ATTTTCTA	41	No	0

ATTCTTCA	2716	2348.17	2896	2465.08	395.248	2529	2255.7	2691	2363.65	289.221	TGAAGAAT	31	No	1.11E-16

Top 25 overrepresented words for the distal promoters in *Arabidopsis thaliana*. The *Word *attribute describes the short nucleotide sequence associated with a putative word. *S *and *ES *describe the number of sequences a word occurs in and the number of sequences the word was expected to occur in respectively, while *O *and *EO *describe the total number of occurrences and the expected total number of occurrences. The score *SlnSES *describes a statistical coverage of the sequences analyzed in the set and is based on a Markov Chain Background Model. Each set of attributes was computed for the masked as well as the unmasked version of the corresponding segment with the emphasis placed on the unmasked version (i.e. sorting of the table based on the unmasked *SlnSES *score).

Further information for the word is provided through its reverse complement (*RevComp*) and the position of the reverse complement in the set of results (*RC_Pos*) as well as a notion describing if the word is a genomic palindrome (*Pal*).

Finally, *PValues *describes a p-value that is assigned in order to provide statistical insight allowing the determination if a word is relevant or was discovered as interesting by random chance.

**Table 8:** The top 25 words in the entire genome

	**Unmasked**					**Masked**					**Unmasked**			
**Word**	**S**	**ES**	**O**	**EO**	**OlnOEO**	**S**	**ES**	**O**	**EO**	**OlnOEO**	**RevComp**	**RC_Pos**	**Pal**	**PValues**

AAAAAAAA	5	5	128631	119310	9675.67	5	5	101229	95334	6073.66	TTTTTTTT	1	No	0

TTTTTTTT	5	5	126533	117302	9585.11	5	5	98883	93091.2	5968.36	AAAAAAAA	0	No	1.67E-15

TATATATA	5	5	58215	49385.7	9575.32	5	5	29264	27159.9	2183.54	TATATATA	2	Yes	3.89E-15

ATATATAT	5	5	59429	53453	6298.28	5	5	30192	29596.8	601.111	ATATATAT	3	Yes	3.00E-15

TAAAAAAT	5	5	14823	11276.3	4053.8	5	5	11492	9148.23	2621.21	ATTTTTTA	5	No	4.44E-16

ATTTTTTA	5	5	14743	11385.1	3810.52	5	5	11392	9219.87	2409.99	TAAAAAAT	4	No	3.33E-16

GAAGAAGA	5	5	30102	26908.7	3375.68	5	5	22784	20523.6	2380.53	TCTTCTTC	7	No	0

TCTTCTTC	5	5	30267	27090.3	3356.11	5	5	23044	20902.7	2247.42	GAAGAAGA	6	No	0

TTTTAAAA	5	5	29354	26314.9	3208.24	5	5	19409	17519.9	1987.46	TTTTAAAA	8	Yes	2.55E-15

AATATATT	5	5	14170	11353.5	3140.06	5	5	11168	10179.5	1035.06	AATATATT	9	Yes	1.11E-16

TTTTCTTT	5	5	31066	28174.8	3034.69	5	5	26876	24423.6	2571.58	AAAGAAAA	11	No	0

AAAGAAAA	5	5	31033	28187.3	2984.8	5	5	26861	24502.1	2469	TTTTCTTT	10	No	1.11E-16

AGAGAGAG	5	5	19376	16630.5	2960.63	5	5	12615	11397.8	1280.05	CTCTCTCT	16	No	1.11E-16

TCTCTCTC	5	5	19179	16519.7	2862.73	5	5	12912	11634.1	1345.64	GAGAGAGA	14	No	4.44E-16

GAGAGAGA	5	5	20064	17413.4	2842.81	5	5	13136	11970.7	1220.21	TCTCTCTC	13	No	1.89E-15

AAGAAGAA	5	5	32397	29731.9	2781.12	5	5	24352	23296.2	1079.35	TTCTTCTT	19	No	0

CTCTCTCT	5	5	18513	15956.1	2751.61	5	5	12312	11212.7	1151.45	AGAGAGAG	12	No	1.11E-16

AGAAGAAG	5	5	26477	24049.7	2545.91	5	5	19161	18013.6	1183.17	CTTCTTCT	20	No	8.88E-16

TTATATAA	5	5	11402	9138.11	2523.66	5	5	9262	8518.12	775.46	TTATATAA	18	Yes	1.11E-15

TTCTTCTT	5	5	32333	29910	2518.58	5	5	24550	23579.9	989.811	AAGAAGAA	15	No	0

CTTCTTCT	5	5	26463	24183.9	2383.23	5	5	19432	18332.3	1132.03	AGAAGAAG	17	No	0

TTTTTCTT	5	5	30561	28331	2315.57	5	5	26516	24717.1	1862.84	AAGAAAAA	22	No	0

AAGAAAAA	5	5	30461	28234.7	2311.9	5	5	26488	24756.8	1790.32	TTTTTCTT	21	No	4.44E-16

TTTGTTTT	5	5	32141	29931	2289.6	5	5	27813	26102.2	1765.71	AAAACAAA	36	No	8.88E-16

Top 25 overrepresented words for the entire genome of *Arabidopsis thaliana*. The *Word *attribute describes the short nucleotide sequence associated with a putative word. *S *and *ES *describe the number of chromosomes a word occurs in and the number of chromosomes the word was expected to occur in respectively, while *O *and *EO *describe the total number of occurrences and the expected total number of occurrences. The score *OlnOEO *describes a statistical overrepresentation of the word in the genome and is based on a Markov Chain Background Model. Each set of attributes was computed for the masked as well as the unmasked version of the corresponding segment with the emphasis placed on the unmasked version (i.e. sorting of the table based on the unmasked *OlnOEO *score).

Further information for the word is provided through its reverse complement (*RevComp*) and the position of the reverse complement in the set of results (*RC_Pos*) as well as a notion describing if the word is a genomic palindrome (*Pal*).

Finally, *PValues *describes a p-value that is assigned in order to provide statistical insight allowing the determination if a word is relevant or was discovered as interesting by random chance.

A detailed analysis of the words identified a minimal overlap between the sets of overrepresented words for the different segments. Specifically, considering the list of top 25 words discovered in any of the six segments (and in the genome wide analysis), 175 words were unique to one specific set, 15 words occurred uniquely in two sets, 7 in three sets, 4 in four sets and none in five sets. Only two words (ATTTTTTA, and AATATATT) were shared in six out of seven sets (neither word was present in the 5'UTR set). Note that the word AATATATT has a significant similarity to the sequence of the TATA-box, a regulatory element that is (1) often found in core promoters and (2) known to contribute to the correct positioning of the core transcriptional machinery [42]. It is conceivable that the absence of AATATATT in the 5'UTR set prevents the initiation of transcription at incorrect sites.

The large differences between the various sets of words provide evidence for the existence of segment-specific signatures. Of additional interest is the uniqueness of the word-based genomic signatures in comparison to the signature for the entire *Arabidopsis *genome. Clearly, the segments' signatures distinguish them from each other and from the entire genome.

In addition to uniquely characterizing each segment, the top words discovered in each data set also have a strong probability of being functional regulatory elements. This argument was strengthened by a functional analysis, which is described later in this section.

### Missing Words

Another interesting component of our word-based signature is the set of words NOT contained within the different segments (see Table 9, 10, 11, &12 and Additional file 8, 9, 10, &11), referred to as *unwords *[43] or *nullomers *[44,45]. The absence of words in particular segments is an interesting phenomenon and may represent negative selection pressure or increased mutation rates specific to these words, or structural constraints on DNA [44]. Thus, the missing word sets, which show unwords and their associated scores, serve as important 'fingerprints' for the segments.

**Table 9:** Words not detected in the 3'UTRs

**#WORD**	**E_S**	**E**
CTAGCAGG	5.98269	6.17391

ACTGCCAG	4.99319	5.1526

CGCCTGAT	4.97776	5.13667

GCGTCCGA	4.52742	4.67187

GGGGTGGC	4.5248	4.66917

ACTCCGCC	4.38831	4.5283

CCCGTTCC	4.25101	4.3866

ACACGCCG	4.21714	4.35165

CCCGCTCA	4.193	4.32673

CTGGGCGT	4.06873	4.19847

GACCTGCG	3.71851	3.83704

GCGCAGTA	3.68699	3.80451

GCACCCGA	3.6084	3.7234

GCACCCTC	3.59671	3.71134

CGCACCCA	3.54333	3.65625

CCGCCGTC	3.53385	3.64646

GGGTCGGC	3.52406	3.63636

GCACGCCT	3.35465	3.46154

GCGCAGCC	3.31181	3.41732

CGTCCGCT	3.28252	3.3871

CTGGCGCC	3.2624	3.36634

GGCGACCT	3.25626	3.36

ATACGCCC	3.18816	3.28972

AGCGCTCC	2.98494	3.08

TAGCGCGG	2.98494	3.08

Top 25 words that were expected to occur in the 3'UTR but are not part of the sequences. Each word is identified through is nucleotide sequence and contains information about the expected number of sequences it was computed to occur in (E_S) as well as the expected number of total occurrences in the set of sequences (E). The words are sorted by their expected sequence occurrence.

**Table 10:** Words not detected in the 5'UTRs

**#WORD**	**E_S**	**E**
GGAACTGC	5.1333	5.40909

GAGGACCC	5.02658	5.29661

GCCCTATA	5.015	5.2844

CCGTACCT	4.98236	5.25

GCGAGTAT	4.94491	5.21053

TATCGCAC	4.83088	5.09034

GGTTGCGG	4.69443	4.94652

GCGGAGTG	4.66421	4.91468

AGTACAGC	4.51745	4.76

GTGCCGAT	4.4368	4.675

GTCCTGGG	4.41572	4.65278

CGGCCGTG	4.3768	4.61176

GGTCGGGG	4.16843	4.39216

GTGCTGGG	4.13122	4.35294

TAGTGCAC	4.12843	4.35

TACCGGCC	4.08277	4.30189

GCCTACGC	4.03144	4.24779

CACCGCGG	3.94494	4.15663

GCGGCGTG	3.90217	4.11155

CGCCTTAG	3.77819	3.98089

CAGCCCAG	3.74709	3.94811

TGAACGGG	3.74703	3.94805

CGTACTGC	3.74638	3.94737

GTGCGCCG	3.68013	3.87755

AGTCCTGG	3.67692	3.87417

Top 25 words that were expected to occur in the 5'UTR but are not part of the sequences. Each word is identified through is nucleotide sequence and contains information about the expected number of sequences it was computed to occur in (E_S) as well as the expected number of total occurrences in the set of sequences (E). The words are sorted by their expected sequence occurrence.

**Table 11:** Words not detected in the Introns

**#WORD**	**E_S**	**E**
CGCGGACA	6.1805	6.4557

CCCGGGAG	4.57278	4.77632

CCGGCCCC	4.46781	4.66667

CGCCCCCC	4.45254	4.65072

GCCCACCG	4.16782	4.35331

GCCGCGGG	3.47686	3.63158

CCGAGGGG	3.34433	3.49315

AAGCGCCC	3.17737	3.31875

CGCCAGCG	2.99188	3.125

CGCTCGCG	2.91507	3.04478

GCGTCGCG	2.8245	2.95017

CCGGCACG	2.48216	2.59259

CCGGGGCG	2.25483	2.35514

CCCGCGCC	2.16189	2.25806

TCGGGCGC	2.11021	2.20408

GCGCACGG	2.02051	2.11039

CGCTCCGC	2.00514	2.09434

CGCGACGC	1.99945	2.0884

TGCGCCCG	1.9539	2.04082

GGTGCGCG	1.92911	2.01493

GCGGGCCC	1.90464	1.98936

CGCGGCGA	1.86163	1.94444

GCGCGACG	1.83299	1.91453

GGGCGGGC	1.79662	1.87654

CCGCCGGG	1.73887	1.81622

Top 25 words that were expected to occur in the introns but are not part of the sequences. Each word is identified through is nucleotide sequence and contains information about the expected number of sequences it was computed to occur in (E_S) as well as the expected number of total occurrences in the set of sequences (E). The words are sorted by their expected sequence occurrence.

**Table 12:** Words not detected in the Core Promoters

**#WORD**	**E_S**	**E**
CGCACACC	5.86109	6.3029

GTCCGAAC	5.46787	5.88

GCCCTATG	5.23895	5.6338

GGACGTCG	4.98873	5.36471

GGCCCTAG	4.47129	4.80822

CGCGAGCG	4.35999	4.68852

GATCCCCC	3.92081	4.21622

GGCCGCAT	3.82028	4.10811

TACCCAGG	3.80429	4.09091

GGCCCCTG	3.67267	3.94937

CGCATCCG	3.66922	3.94565

CACGCCGA	3.56933	3.83824

CCGGCCGC	3.51312	3.77778

CGCGGTCA	3.51079	3.77528

AGGGCCCT	3.50922	3.77358

GGCGCTGT	3.49296	3.7561

ACGCCCTG	3.45587	3.71622

GCGGACAC	3.30648	3.55556

AGTGGCGC	3.29952	3.54808

GGGCGTTC	3.26995	3.51628

CGCGCAAG	3.25481	3.5

ACCCGCGT	3.22635	3.46939

TTACCCCG	3.22482	3.46774

CCGGTGCG	3.18249	3.42222

TAGGGCCG	3.18249	3.42222

Top 25 words that were expected to occur in the core promoters but are not part of the sequences. Each word is identified through is nucleotide sequence and contains information about the expected number of sequences it was computed to occur in (E_S) as well as the expected number of total occurrences in the set of sequences (E). The words are sorted by their expected sequence occurrence.

### Word-based Clusters

Any biologically required sequence experiences evolutionary pressure (in this case purifying selection) resulting in a narrowing of the range of allowable sequence mutations. Often, a word and various mutations of the word exhibit the same functionality. To incorporate this into our analysis, clusters were built around each of the top overrepresented words, forming groups of words that are similar to each 'seed word.' Word similarity was measured through the Hamming distance metric, which models single point mutations. A Hamming distance of 1 was used to form the clusters. Each cluster is depicted via a sequence logo, providing a visual motif of the characteristics of the cluster.

Selected clusters and the corresponding sequence logos are shown in Additional file 12. Two representative motifs are presented for each segment. Motifs for each segment were chosen in order to provide a variety of examples of putative binding sites for the non-coding segments.

The presented motifs correspond to well-known regulatory elements and complex motifs, which represent sets of putative regulatory elements. Of particular interest in Additional file 12 are the word-based clusters for the core promoters (in the left column) which correspond to the TATA-box. Also known as the Goldberg-Hogness box [46], the TATA-box is a well-characterized regulatory element appearing 31 bp upstream of the transcription start site in 30% of the promoter sequences in *Arabidopsis *[23]. The core promoters also contain another interesting motif, (CGACGTCG), which is involved in stress response in *Arabidopsis thaliana *[22]. An extensive functional characterization is described later in this section.

### Word Location Distribution

**Figure 1:**
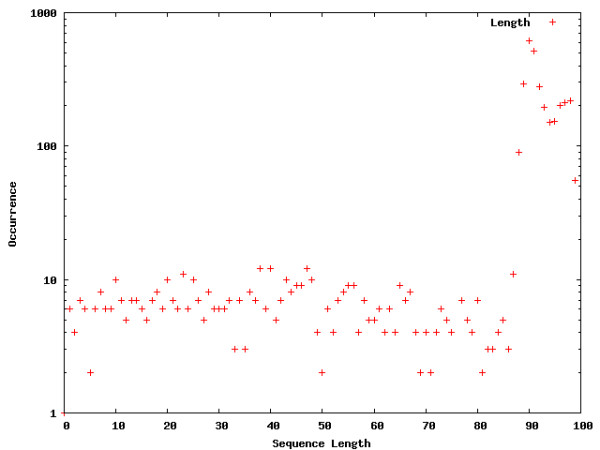
**Word location distribution across introns**. Word location distributions for interesting words within the introns. The occurrences are shown on a log-scale in order to allow a comparison between the different segments as well as the words visualized for the entire genome.

**Figure 2:**
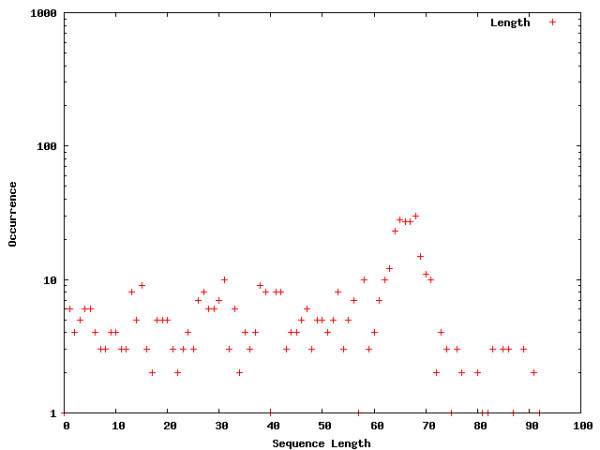
**Word location distribution across core promoters**. Word location distribution for interesting words within the core promoters. The occurrences are shown on a log-scale in order to allow a comparison between the different segments as well as the words visualized for the entire genome.

**Figure 3:**
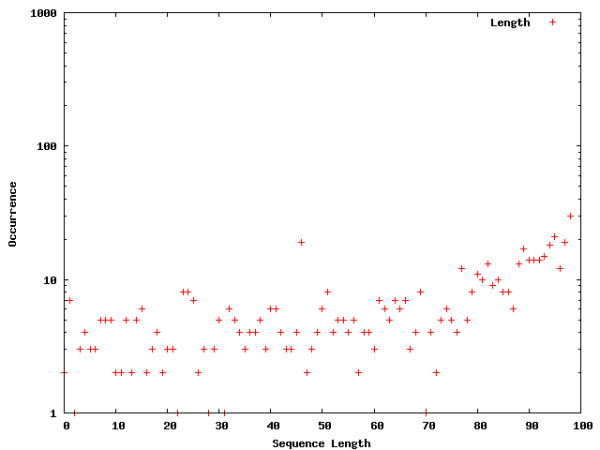
**Word location distribution across proximal promoters**. Word location distributions for interesting words within proximal promoters. The occurrences are shown on a log-scale in order to allow a comparison between the different segments as well as the words visualized for the entire genome.

**Figure 4:**
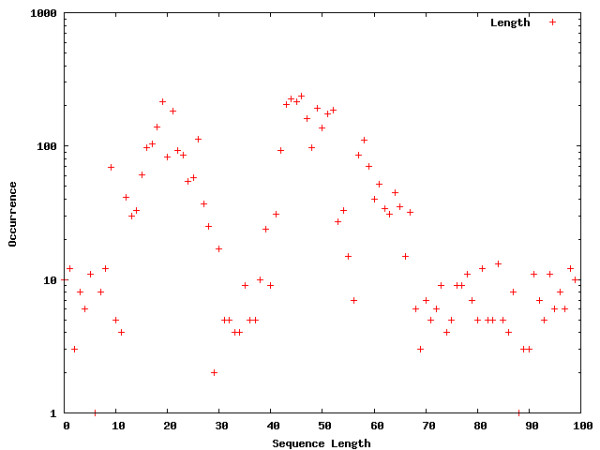
**Word location distribution across the entire genome**. Word location distributions for interesting words within the genome. The occurrences are shown on a log-scale in order to allow a comparison between the different segments as well as the words visualized for the entire genome.

The locations of a particular word within a segment can provide insight into functional properties of the word. For example, functional TATA motifs are located at a specific distance upstream of the TSS [23,46]. We identified the segment-specific locations of the seed words of the clusters shown in Additional file 12. Being selected for their high complexity, these words are expected to exhibit a distribution bias, manifesting as peaks in the scatterplots of the distribution across sequences, as shown in Figures 1, 2, 3 and 4.

The Figures contain histograms showing the numbers of occurrences of specific words at each point along the sequences. For uniformity, sequence lengths are normalized to the range [1;100]. Strong peaks can indeed be found for the words selected in the intron, core promoter, and proximal promoter regions. The peaks detected for the intron segment are at both the 5' and 3' ends of the introns, which means that the words occur in close proximity to flanking exons. The close proximity to the intron-exon boundaries is expected for splicing regulatory sequences [2,8-16]. The peaks exhibited in core and proximal promoters are not surprising. The distributions of words locations in these segments are expected to show clustering, due to positional conservation of locations of *cis*-regulatory elements [23]. Of particular interest is the location of the peak for the first word chosen for the core promoter distribution (TATAATA), the TATA-box. A location of around 31 bp upstream from the TSS corresponds to the findings in [23].

Interestingly, we also detect strong peaks for the example words chosen for the genome wide word landscape, possibly indicating an important chromosomal feature that is not yet understood.

### Word Co-occurrences

Genes are usually controlled by a combination of multiple transcription factors, or by transcription factor complexes binding to different sites embedded in the genes' regulatory non-coding regions. In order to detect the interacting transcription factor binding sites of a complex, we examined the positional relationships of words. The top 25 overrepresented words were paired, and the overrepresentation of each pair was determined using a Markovian background model of order 6. The top 25 overrepresented word pairs for each segment are displayed in Table 13, 14, 15, 16, 17 and 18 (see also Additional files 13, 14, 15, 16, 17, &18). The limited overlap between the word pairs of different segments indicates additional unique word-based signatures for genomic segments.

**Table 13:** Co-occurrence in 3'UTRs

**Word1**	**Word2**	**S**	**E_S_**	S*ln(S/E_S_)
TTCTTTTT	TTTTTCTT	322	238.5802	96.5504

TGTTTTTT	TTTTTCTT	283	217.7183	74.2154

TTCTTTTT	TTTTTTCT	260	197.5705	71.3925

TTTTTCTT	TTTTTGTT	326	273.0848	57.7395

TCTTTTTT	TTTTTCTT	270	218.9471	56.5898

TTTTCTTT	TTTTTTCT	278	226.8886	56.479

TTTTTTGG	TTTTTGTT	161	116.5969	51.9517

TTATTTTT	TTTTTCTT	211	166.8299	49.5604

TTCTTTTT	TTTTTGTT	290	248.3755	44.9324

TGTTTTTT	TTCTTTTT	239	198.0677	44.8973

TTTTCTTT	TCTTTTTT	270	228.7449	44.7699

TCTTTTTC	TTTTTTCT	112	76.7939	42.2658

TGTTTTTT	TTTTTTGG	129	93.1111	42.0564

TTTTTTGG	TTTTTCTT	148	112.0287	41.2117

TTTTTTCT	TTTTTTGG	128	92.8787	41.0542

TTTTCTTT	TGTTTTTT	265	227.4605	40.4796

TTTGTTTT	TTTTTTGG	170	134.4256	39.9138

TTCTTTTT	TTTTTTGG	136	101.9687	39.1665

TCTTTTTT	TTTTTTGG	127	93.6332	38.7099

TTTTCTTT	TTCTTTTT	285	249.2674	38.1794

TTTTTATT	TTATTTTT	137	103.7794	38.0467

TGTTTTTT	TTTTTTCT	215	180.3272	37.8109

TCTTTTTT	TTTTTTCT	216	181.3431	37.7758

TTTTTGGT	TTTTTGTT	161	127.4072	37.6766

ATTTTTTA	TTTTTCTT	82	53.2457	35.4078

Overrepresented non-overlapping word-pairs detected in the 3'Untranslated Regions of *Arabidopsis thaliana*. A word-pair is characterized through the two nucleotide sequences associated with it (Word1 and Word2), the number of sequences the pair occurs in (S) as well as the expected number of sequences (E_S_) and a statistical score symbolizing the overrepresentation of the word-pair in the specific sequence set (S*ln(S/E_S_)).

**Table 14:** Co-occurrence in 5'UTRs

**Word1**	**Word2**	**S**	**E_S_**	S*ln(S/E_S_)
CTCTCTTT	CTTTCTCT	209	108.1185	137.7533

TTTCTCTC	CTCTCTTT	214	139.4419	91.6622

TTTCTCTC	CTTTCTCT	198	125.808	89.7949

TTTTTTGT	TTTTCTTT	97	41.7516	81.7683

CTTCTCTT	CTCTTCTC	97	45.9973	72.3745

CTCTGTTT	TTTTTCTT	105	54.0587	69.7085

TTTTTTGT	TTTTTCTT	97	48.6186	66.9983

TTTTCTTT	TTTTTCTT	122	71.3728	65.4048

TTTTTGTT	TTTTTCTT	115	65.2326	65.2019

TTTCTCTC	CTCTTCTC	128	78.07	63.2863

TTTTCTTT	TTTTTGTT	103	56.0093	62.7487

CTCTGTTT	TTTTTGTT	87	42.4337	62.4629

AAAGAAAA	AGAAAAAA	130	82.9236	58.4498

CTCTCTGT	CTTTCTCT	90	47.3124	57.8733

CTTTCTCT	CTCTTCTC	105	60.5869	57.7376

TTTTCTCC	CTCTTCTC	61	23.918	57.1107

ACAAAAAA	AAAAAACA	92	49.5364	56.9554

CTTTCTTC	CTCTTCTC	88	47.0073	55.179

AAGAAAAA	AGAAAAAA	141	95.4769	54.9724

CTCTCTTT	CTCTTCTC	109	67.1219	52.8472

GAAAGAGA	AGAGAAAG	57	22.6518	52.6003

TTTCCTCT	CTTTCTCT	79	40.6193	52.5511

TTTCCTCT	TTTCTCTC	91	52.3194	50.3678

TTTTCTTT	CTCTCTTT	127	85.6598	50.013

TTCTCTCC	CTCTTCTC	53	21.4631	47.9097

Overrepresented non-overlapping word-pairs detected in the 5'Untranslated Regions of *Arabidopsis thaliana*. A word-pair is characterized through the two nucleotide sequences associated with it (Word1 and Word2), the number of sequences the pair occurs in (S) as well as the expected number of sequences (E_S_) and a statistical score symbolizing the overrepresentation of the word-pair in the specific sequence set (S*ln(S/E_S_)).

**Table 15:** Co-occurrence in Introns

**Word1**	**Word2**	**S**	**E_S_**	S*ln(S/E_S_)
TTTTATTT	ATTTTTTA	393	217.8144	231.9354

TTTTTATT	ATTTTTTA	334	186.0726	195.3914

TAAAAAAT	AATATATT	147	39.3119	193.8792

TTTTTAAT	TTTTTATT	460	306.2869	187.084

TAAAAAAT	TTTTATTT	273	140.3538	181.6284

TAATTTTT	ATTTTTTA	238	113.2939	176.6639

CTCTGTTT	CTGTTTTT	346	208.3136	175.5583

TTTTATTT	AATATATT	308	175.8151	172.6854

TTTTATTT	TTTTTAAT	505	358.7745	172.6415

TAAAAAAT	ATTTTTTA	149	48.6332	166.8264

TAAAAAAT	TTTTTAAT	189	79.759	163.0573

TAAAAAAT	TAATTTTT	179	73.1119	160.2756

TTTTATTT	TAATTTTT	461	328.5857	156.0948

TTTTTAAT	ATTTTTTA	238	123.6151	155.9133

TAAAAAAT	TTTTTCTT	305	185.7949	151.1788

TAAAAAAT	TTTTTATT	230	119.9486	149.7338

TTTTTATT	AATATATT	261	150.2261	144.1709

TAATTTTT	TTTTTAAT	300	186.1617	143.1501

TTTTTAAT	AATATATT	202	99.8493	142.3303

TTTTATTT	TTTTTATT	670	542.1648	141.8441

TAAAAAAT	TTTTTTGT	262	157.163	133.898

TAATTTTT	AATATATT	187	91.5206	133.6198

ATTTTTTA	TTTTTTGT	354	243.9756	131.769

TAAAAAAT	TTTTGTTT	357	246.9371	131.5909

TTTTTAAT	TTTTTGTT	638	519.9558	130.5312

Overrepresented non-overlapping word-pairs detected in the introns of *Arabidopsis thaliana*. A word-pair is characterized through the two nucleotide sequences associated with it (Word1 and Word2), the number of sequences the pair occurs in (S) as well as the expected number of sequences (E_S_) and a statistical score symbolizing the overrepresentation of the word-pair in the specific sequence set (S*ln(S/E_S_)).

**Table 16:** Co-occurrence in Core Promoters

**Word1**	**Word2**	**S**	**E_S_**	S*ln(S/E_S_)
GCCCAATA	GCCCATTA	32	2.3492	83.5729

TTTTTTCT	TTTTTCTT	68	22.9531	73.8516

AATAAAAA	AAGAAAAA	84	41.5798	59.069

CTCTCTTT	CTTTCTCT	40	9.1626	58.95

AATAAAAA	ATTAAAAA	57	22.4453	53.1222

ACAAAAAA	AAGAAAAA	71	35.1265	49.9645

ACAAAAAA	AGAAAAAA	66	31.1075	49.6455

ATTTCTCA	TATAAATA	30	6.1031	47.772

AATAAAAA	TAAAAAAT	38	10.8748	47.5432

AAAAAACA	ACAAAAAA	56	24.4921	46.3121

AAAAATAT	AAAAAACA	44	15.5191	45.8533

AACAAAAA	AAGAAAAA	77	42.5433	45.6828

AACAAAAA	AGAAAAAA	69	37.6758	41.7512

TTTCTTTT	TTTTTTGT	40	14.2927	41.1653

AAAAAACA	ATATAAAG	30	7.659	40.9596

AAAAAACA	CTATATAA	36	11.9538	39.689

AAAAATAT	CTATATAA	30	8.0863	39.3309

TATATAAA	TAAAAAAT	36	12.3623	38.4793

AATAAAAA	TTAAAAAA	53	25.8324	38.0892

TTTTATTT	TTTTTTAA	38	14.0039	37.9336

TTTTATTT	TTTTTCTT	50	23.5743	37.5932

TTCTTTTT	TTTTTCTT	46	20.3942	37.416

AAATTAAA	ACAAAAAA	44	18.9721	37.0137

AATAAAAA	AGAAAAAA	65	36.8225	36.938

TTTCTTTT	TTTTTGTT	41	16.8429	36.4755

Overrepresented non-overlapping word-pairs detected in the core promoters of *Arabidopsis thaliana*. A word-pair is characterized through the two nucleotide sequences associated with it (Word1 and Word2), the number of sequences the pair occurs in (S) as well as the expected number of sequences (E_S_) and a statistical score symbolizing the overrepresentation of the word-pair in the specific sequence set (S*ln(S/E_S_)).

**Table 17:** Co-occurrence in Proximal Promoters

**Word1**	**Word2**	**S**	**E_S_**	S*ln(S/E_S_)
AAATTTTA	TAAAAAAT	996	489.8445	706.8206

ATTTTTTA	TAAAAAAT	869	395.77	683.4771

TAAATTTT	TAAAAAAT	970	501.8706	639.1852

AAAAATTA	TAAAAAAT	1040	565.2386	634.1171

TAAAATTT	TAAAAAAT	963	498.7952	633.5171

TAAAATTT	ATTTTTTA	892	458.4645	593.7003

AAATTTTA	ATTTTTTA	868	450.2375	569.7695

AAAAATTA	ATTTTTTA	947	519.5356	568.5445

AAAATTTA	TAAAAAAT	919	496.1801	566.4231

TAATTTTT	TAAAAAAT	965	539.2575	561.5671

AAAATTTA	ATTTTTTA	865	456.0608	553.6894

TAATTTTT	ATTTTTTA	907	495.6552	548.0656

AATATATT	TAAAAAAT	776	391.8276	530.2646

AAAATTTA	AAATTTTA	973	564.4665	529.8015

AAATTTTA	TAAAATTT	976	567.4415	529.3092

AAAAATTA	TAATTTTT	1125	707.8947	521.1483

AATATATT	ATTTTTTA	730	360.1459	515.7708

TAAATTTT	ATTTTTTA	845	461.2912	511.4845

AAAAATTA	TAAAATTT	1052	654.7789	498.8066

AAAATTTA	AAAAATTA	1044	651.346	492.5318

AAAATTTA	TAAAATTT	958	574.7807	489.4031

AAATTTTA	TAATTTTT	993	613.4724	478.2242

TAATTTTT	TAAAATTT	995	624.6821	463.1724

AAAATTTA	TAATTTTT	990	621.407	461.0615

TTATATAA	TAAAAAAT	645	316.3233	459.5531

Overrepresented non-overlapping word-pairs detected in the proximal promoters of *Arabidopsis thaliana*. A word-pair is characterized through the two nucleotide sequences associated with it (Word1 and Word2), the number of sequences the pair occurs in (S) as well as the expected number of sequences (E_S_) and a statistical score symbolizing the overrepresentation of the word-pair in the specific sequence set (S*ln(S/E_S_)).

**Table 18:** Co-occurrence in Distal Promoters

**Word1**	**Word2**	**S**	**E_S_**	S*ln(S/E_S_)
TAAAAAAT	ATTTTTTA	1855	898.8038	1344.087

AATATATT	TAAAAAAT	1759	902.7094	1173.429

AATATATT	ATTTTTTA	1692	882.8679	1100.631

TTATATAA	ATTTTTTA	1478	740.7429	1020.99

TTATATAA	TAAAAAAT	1464	757.3903	964.8477

AATATATT	TTATATAA	1447	743.9616	962.6287

AAAAATTG	TAAAAAAT	1301	747.7933	720.4442

CAATTTTT	TAAAAAAT	1279	745.3293	690.6698

AAAAATTG	ATTTTTTA	1237	731.3568	650.0966

ATTTTGTA	ATTTTTTA	1156	665.4975	638.3272

CAATTTTT	ATTTTTTA	1200	728.947	598.171

TAGAAAAT	TAAAAAAT	1024	586.114	571.3484

ATTTTGTA	TAAAAAAT	1108	680.4539	540.2074

CAATTTTT	AATATATT	1162	732.1145	536.7987

ATTTTTCA	ATTTTTTA	1078	666.4705	518.3745

AAAAATTG	AATATATT	1148	734.5348	512.627

CAATTTTT	TTATATAA	1003	614.2579	491.8069

TAGAAAAT	AATATATT	956	575.7221	484.8189

ATTTTCTA	ATTTTTTA	952	574.2477	481.2399

ATTTTCTA	TAAAAAAT	964	587.1534	477.9562

TAGAAAAT	ATTTTTTA	941	573.2313	466.4103

ATTTTTCA	TAAAAAAT	1058	681.4487	465.4297

TGAAAAAT	ATTTTTTA	1020	658.2655	446.7086

TGAAAAAT	TAAAAAAT	1033	673.0593	442.5259

AAAAATTG	TTATATAA	970	616.2886	439.9733

Overrepresented non-overlapping word-pairs detected in the distal promoters of *Arabidopsis thaliana*. A word-pair is characterized through the two nucleotide sequences associated with it (Word1 and Word2), the number of sequences the pair occurs in (S) as well as the expected number of sequences (E_S_) and a statistical score symbolizing the overrepresentation of the word-pair in the specific sequence set (S*ln(S/E_S_)).

### AGRIS Lookup

The AGRIS database [25] contains a large collection of known regulatory elements for *Arabidopsis thaliana*. The words discovered in this study were compared to the regulatory elements of equal or lesser length in AGRIS. Table 19 provides the overview of the motifs and their locations within the results.

**Table 19:** AGRIS Lookup

	**3'UTRs**		**5'UTRs**		**Intron**		**Core Promoters**		**Proximal Promoters**		**Distal Promoters**	
**Regulatory Element from AGRIS database **[25]	**Rank**	**Score**	**Rank**	**Score**	**Rank**	**Score**	**Rank**	**Score**	**Rank**	**Score**	**Rank**	**Score**
Bellringer/replumless/pennywise BS3 IN AG	-	-	-	-	43503	0.0479784	-	-	64618	0.955909	56341	-103.557

CBF1 BS in cor15a	-	-	-	-	48346	-1.48116	-	-	4852	1.34988	11624	24.1708

Octamer promoter motif	-	-	-	-	41435	0.673899	-	-	11935	1.28979	23858	4.69741

Bellringer/replumless/pennywise BS1 IN AG	72	67.6311	352	35.2087	574	127.468	19	117.144	875	1.0759	58337	-186.12

ABRE-like binding site motif	5445	11.7462	1138	21.7556	15242	16.0488	304	41.9698	53	1.45099	109	255.929

G-box promoter motif	1852	21.1577	1138	21.7556	12023	20.8282	304	41.9698	53	1.45099	102	260.604

DPBF1&2 binding site motif	3720	14.7278	2963	13.7441	3460	54.8094	355	39.8827	137	1.36496	102	260.604

MYB1 binding site motif	4306	13.6223	446	32.0594	1407	86.7638	400	38.3647	1785	1.11027	2557	76.5745

RAV1-A binding site motif	568	34.0603	148	49.0095	2000	73.6726	451	36.3111	135	1.20169	289	186.355

W-box promoter motif	751	30.7769	675	27.0198	458	139.172	533	34.175	176	1.19182	756	131.24

CBF2 binding site motif and GBF1/2/3 BS in ADH1	-	-	-	-	34949	2.87187	540	34.0562	729	1.293	998	117.554

ARF and ARF1 binding site motif	976	27.5809	216	42.5544	741	116.214	568	33.5619	2852	1.07934	2306	80.856

L1-box promoter motif	2697	17.6326	-	-	5824	38.2912	585	33.083	2889	1.05367	2235	81.9035

GATA promoter motif	1186	25.6353	741	26.1103	1247	91.6715	802	29.355	355	1.08161	1033	115.612

ATB2/AtbZIP53/AtbZIP44/GBF5 BS in ProDH	1757	21.6648	1225	20.9254	2890	60.5806	908	27.9139	1313	1.12688	3204	67.6808

SORLIP2	3658	14.8663	9024	6.91197	16361	14.6754	1006	26.5383	550	1.34186	780	129.375

MYB binding site promoter	4762	12.8183	2462	15.1743	1897	75.734	1032	26.1692	4931	1.06605	2010	86.739

CCA1 binding site motif	1230	25.1325	371	34.5029	5202	41.532	1225	24.4536	61990	0.99765	58013	-161.161

TGA1 binding site motif	-	-	13290	4.96662	10326	24.0526	1233	24.3919	1660	1.21323	1879	89.7072

SORLIP1	5297	11.9625	6172	9.0064	11076	22.5348	1286	23.8899	4965	1.15533	4097	58.1886

T-box promoter motif	639	32.6567	1532	19.0267	774	114.265	1325	23.5609	193	1.27522	205	212.153

Ibox promoter motif	2156	19.649	358	35.0463	3223	57.1901	1797	20.4507	1081	1.14622	628	140.679

Box II promoter motif	1403	23.9863	4993	10.3195	1437	85.6577	1804	20.4254	1986	1.30314	669	136.891

Hexamer promoter motif	7590	9.4166	1616	18.5991	10347	24.0156	2225	18.6568	3477	1.24419	1252	107.567

AtMYC2 BS in RD22	1193	25.5614	4026	11.6309	3460	54.8094	2823	16.6193	646	1.21499	2073	85.133

RAV1-B binding site motif	7054	9.94571	8250	7.4051	11589	21.6087	2996	16.0975	6084	1.12709	2017	86.5658

RY-repeat promoter motif	182	49.4382	-	-	530	132.253	3097	15.8378	72	1.29305	61	302.629

MYB3 binding site motif	5128	12.2348	10575	6.06616	1407	86.7638	3292	15.3953	3288	1.08324	11546	24.3649

Bellringer/replumless/pennywise BS2 IN AG	3126	16.2923	-	-	64424	-30.4349	3694	14.5011	62777	0.97976	58184	-172.62

AtMYB2 BS in RD22	6797	10.1949	9630	6.55608	4961	42.997	4480	13.0383	3570	1.07359	3218	67.5209

E2F binding site motif and E2F/DP BS in AtCDC6	-	-	4078	11.5443	46644	-0.929602	4953	12.223	60966	1.20703	55143	-85.466

ERF1 BS in AtCHI-B and GCC-box promoter motif	-	-	681	26.9446	20822	10.4265	6359	10.5016	4340	1.35349	1735	93.0802

Z-box promoter motif	-	-	-	-	36029	2.48082	10144	7.62515	39199	1.00107	26784	1.42726

LTRE promoter motif	-	-	6230	8.95512	16036	15.0374	11248	7.01938	11296	1.13624	7155	38.6247

SORLIP5	5170	12.1706	3175	13.3137	14017	17.6817	11614	6.82909	14984	1.04471	22267	6.5221

ABFs and ABRE binding site motif	8540	8.6035	6266	8.92287	29109	5.33319	12250	6.52158	725	1.25598	1490	100.349

PI promoter motif	9436	7.96403	-	-	60410	-9.96838	14596	5.56209	24540	1.01231	7902	35.621

Observations about the regulatory elements (length = 8) contained in the AGRIS database [25].

### Functional Categorizations of Words

In order to reveal biological meanings of overrepresented words, we established associations between the overrepresented words and biological functions of the genes that harbour a particular word in their corresponding segment (Table 1). For a single word, all the genes that contain that word in their selected segment were found and the corresponding overrepresented Gene Ontology (GO) terms were identified. Overrepresentation of a GO term is determined by using the *Arabidopsis *gene GO term distributions as a background model. The developmental and experimental parameters that perturb the expression of genes harbouring a particular word was determined by comparing the number of induced, suppressed or neutral genes, to that expected by chance in a collection of 1305 tissue and stress microarrays from the public domain. Significant enrichment or depletion of induced or suppressed genes has been shown to correlate strongly with factors affecting regulation of a *cis*-regulatory element [39].

**Figure 5:**
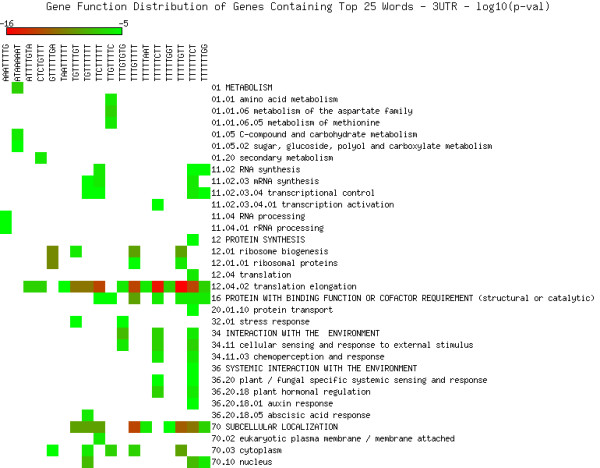
**Cellular functions in 3'UTRs**. Enriched functional categories within the set of genes associated with each word in the top 25 words of the 3'UTRs. The lookup was conducted against the MIPS Functional Catalogue Database (FunCatDB) [54].

**Figure 6:**
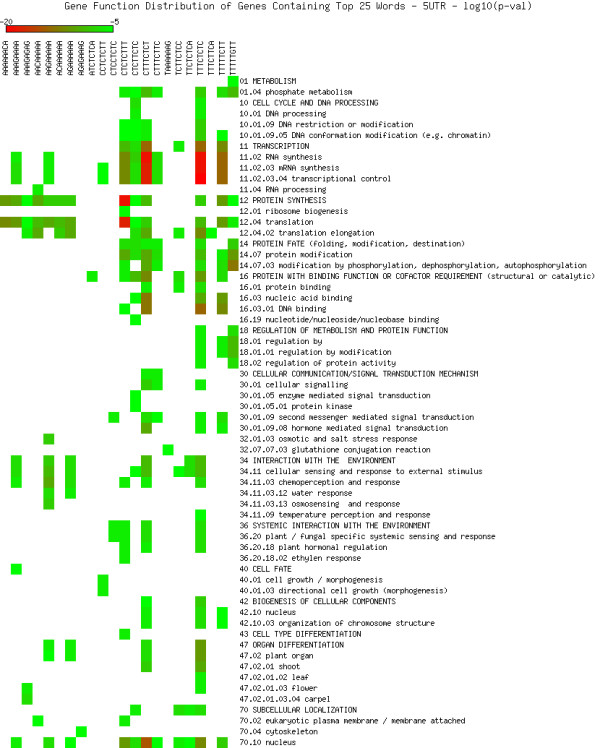
**Cellular functions in 5'UTRs**. Enriched functional categories within the set of genes associated with each word in the top 25 words of the 5'UTRs. The lookup was conducted against the MIPS Functional Catalogue Database (FunCatDB) [54].

**Figure 7:**
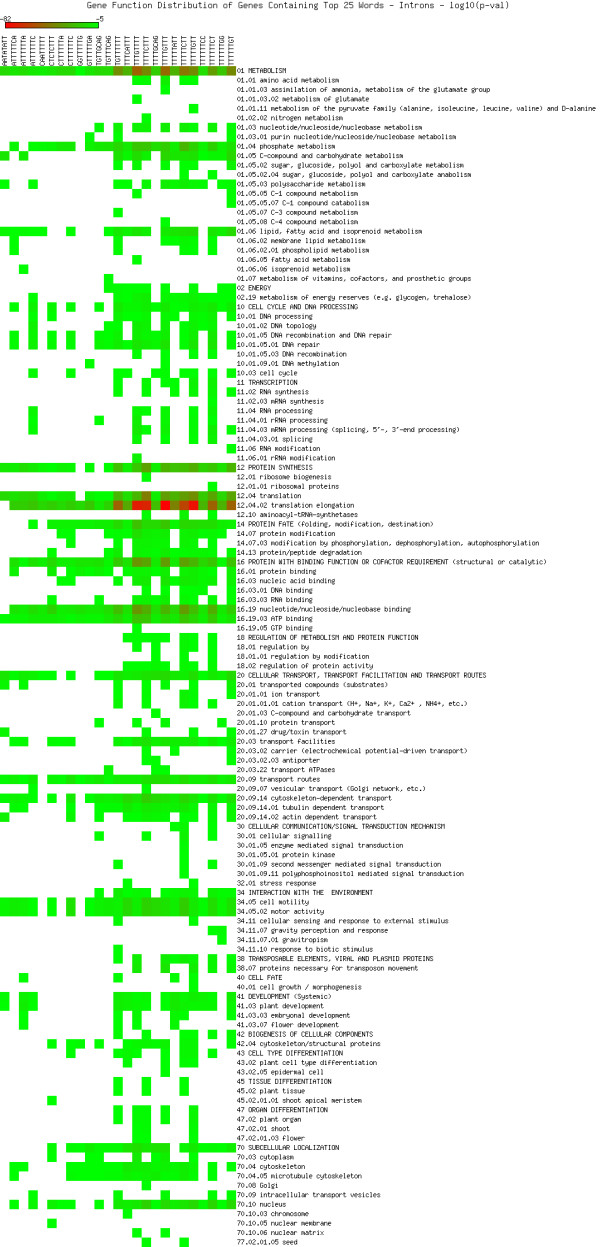
**Cellular functions in introns**. Enriched functional categories within the set of genes associated with each word in the top 25 words of the introns. The lookup was conducted against the MIPS Functional Catalogue Database (FunCatDB) [54].

**Figure 8:**
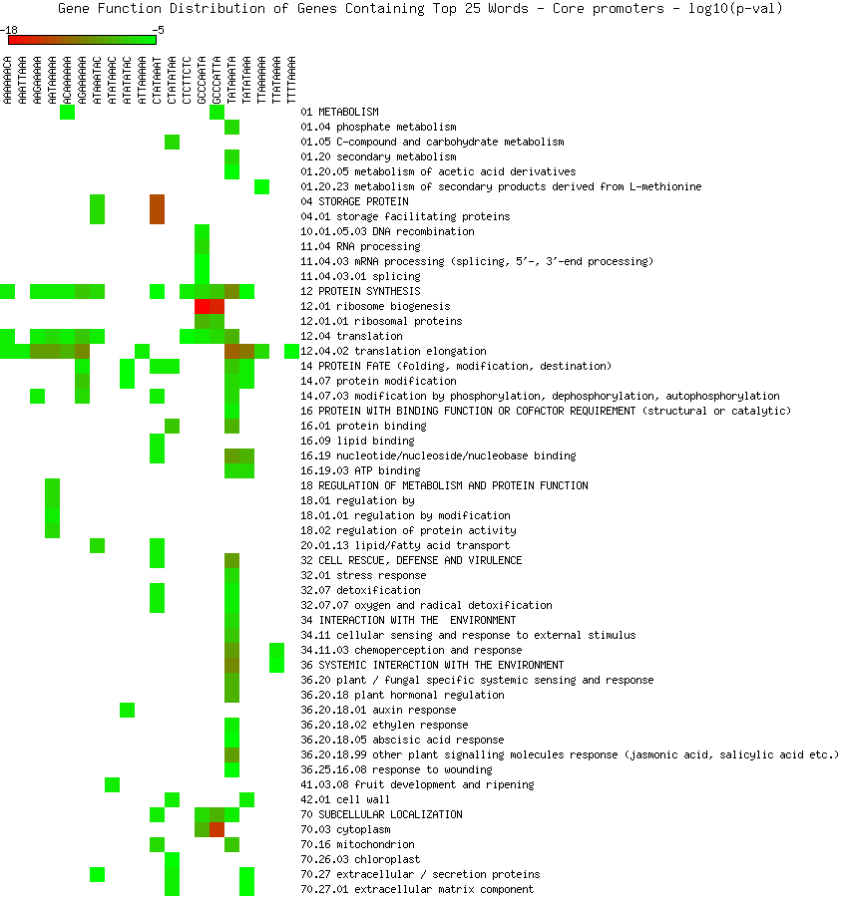
**Cellular functions in core promoters**. Enriched functional categories within the set of genes associated with each word in the top 25 words of the core promoters. The lookup was conducted against the MIPS Functional Catalogue Database (FunCatDB) [54].

**Figure 9:**
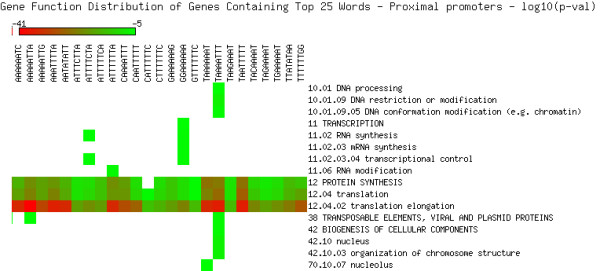
**Cellular functions in proximal promoters**. Enriched functional categories within the set of genes associated with each word in the top 25 words of the proximal promoters. The lookup was conducted against the MIPS Functional Catalogue Database (FunCatDB) [54].

**Figure 10:**
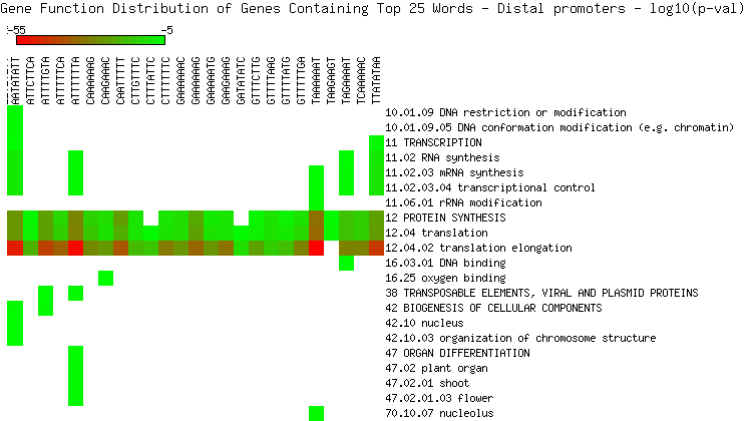
**Cellular functions in distal promoters**. Enriched functional categories within the set of genes associated with each word in the top 25 words of the distal promoters. The lookup was conducted against the MIPS Functional Catalogue Database (FunCatDB) [54].

As shown in Figures 5, 6, 7, 8, 9 and 10, we identified overrepresented functional categories (y-axis) of genes that carry a particular word (x-axis, top panel) in either their 3'UTR (Figure 5), 5'UTR (Figure 6), intron (Figure 7), or promoter regions (Core, Proximal and Distal Promoters, Figures 8, 9 and 10, respectively). The red squares depict overrepresented categories with lowest p-value, calculated for each segment separately, smaller than 10E-16. For example, the word GTTTTTGA was significantly enriched in the 3'UTRs of genes that participate in the GO category "*Protein Synthesis" *(including the sub-categories ribosome biogenesis, ribosomal proteins, translation), and is correlated with genes suppressed in flowers and early stage siliques (p-value 4E-14). Based on microarray expression of micro-dissected tissues (see methods), the word TGTTTTTT is present in the 3' UTR of genes induced in roots (p-value 1E-8), in the atrichoblast (hairless) cell files of the root (p-value 7E-25), the root cortex (p-value 2E-23), endodermis (p-value 2E-51), and lateral root cap (p-value 4E-20). The word CTCTCTTT, enriched in introns, was correlated with differential induction in cotyledons (p-value 8E-20), suppressed in young flowers, especially carpals (p-value 1E-14) and heart stage embryos (p-value 3E-20). Surprisingly, the presence of these words in the UTRs and introns were strongly correlated with tissue specific profiles, but were only weakly enriched or strongly depleted for responses by hormones, biotic and abiotic stresses. There was no significant correlation to any of the 1305 surveyed conditions if the words were located in the 1000 bp upstream or downstream regions. This is strikingly different to the well characterized abscisic acid responsive element (ABRE) (CACGTGTC) [22], which when found in the 1000 bp 5'upstream region, was strongly correlated to induction by 10 μM abscisic acid (ABA) (p-value 4E-49), cold, salt and drought stresses (p-values < 1E-40), in flowers (p-value 1E-31), and suppressed in roots (p-value 4E-7) but no significant correlations were observed when ABRE was present in the 3'UTRs, 5'UTRs or introns. We also analyzed primary promoter regions where most of the basal promoter elements are expected to be located. The frequency of words is calculated as described above, and genes that contain the high scoring word in their primary promoter region were queried for enriched biological function. For example, GCCCATTA is found in core promoter regions of genes preferably involved in ribosome biogenesis and translation. Genes with this word in the upstream promoter are significantly depleted for response to all hormones, biotic and abiotic stresses (typically p-value 1E-8 or better). In other words, genes harbouring this word in their upstream promoter region tend to be less responsive to stresses than randomly chosen genes. However, the word CTATAAAT was found in core promoter regions of genes preferably functioning as storage facilitating proteins (Figure 8). Genes with this word in the upstream promoter are rapidly induced by 10 nM brassinolide (p-value 1E-9) and by salt stress in roots (p-value 4E-9). These genes are also induced in roots, flowers, pollen, and during seed development, and strongly suppressed in young leaves and cotyledons.

A set of 10 frequently enriched cis-elements was recently provided for the ATH95 gene coexpression neighbourhood (AAACCCTA, CTTATCCN, GGCCCANN, GCCACGTN, GCGGGAAN, GACCGTTN, AANGTCAA, CNGATCNA, NCGTGTCN, CATGCANN) [47]. Our results show a direct overlap with two of those words (AAACCCTA, NCGTGTCN), which are detected and marked as 'interesting' in the 5'UTRs, and the proximal promoters, respectively. Several words were hit partially as members of the 'interesting' word clusters (CTTATCCN, GCCACGTN, AANGTCAA, CNGATCNA), while others were not represented in the selected word clusters and the top 25 words. While no overlap for GACCGTTN could be found, it is possible to validate the significance of GGCCCANN and GCGGGAAN through the detection of these two words as unwords in the introns, marking them interesting regulatory elements associated with the expression, but not necessarily with the regulation of the associated alternative splicing process.

## Conclusion

The analyses described here provide a first view of the word landscape within the non-coding regions of the *Arabidopsis thaliana *genome. An analysis centred on the statistically interesting words furnishes important insights into the unique elements of each segment. The correlations of particular words with cellular functions or expression patterns provide valuable hypotheses for further experimentation. Correlation between word position and expression also seems strong, with one class of words only present in the 5'/3'UTRs and introns, and another class of words putatively functioning only in the region upstream of the TSS. Words in the first class seem more directed at regulation of tissue and cellular identity, while words which function upstream appear more likely to be involved in environmental responses.

## Methods

**Figure 11:**
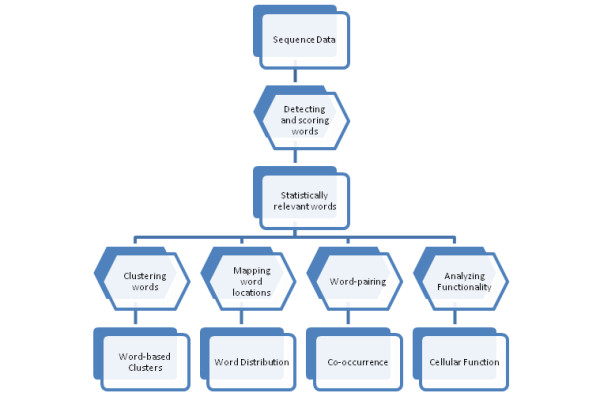
**Process Flowchart**. Methodology flow applied for the discovery of word-based genomic signatures in non-coding *Arabidopsis thaliana*.

Word-based genomic signatures are the union of results generated by applying the software pipeline shown in Figure 11. Statistically relevant words are extracted from a set of genomic sequences, and are analyzed to determine similarity, location distribution, groupings, and predicted cellular function.

### Sequence Data

This manuscript reports the results of analyzing DNA sequences of *Arabidopsis thaliana*. The non-coding genomic segments (specifically, the 3'UTRs, 5'UTRs, promoters and introns) and the entire genomic sequence (as complete chromosomes) were obtained from TAIR (release 8) [19]. Both masked and unmasked versions of the genome were analyzed. Ambiguous nucleotides, depicted in the sequences by the letters [R, Y, W, S, K, M], were removed because they represent sequencing anomalies; this resulted in the removal of 0.15% (or 188,820) of the nucleotides.

In this study, only protein-coding genes were considered as genes, and transposable-like, or pseudo-genes, were omitted. Thus, the total number of genes in this study is ~27,000. Due to different lengths and locations of the promoter elements it is possible that, while core promoters can occur for a specific gene, no distal promoter for that gene exists due to the fact that its location would fall into another gene or even outside of a chromosome. The difference in number of genes in 3'UTRs and 5'UTRs sets compared to other sets is due to genes that lack annotated UTR (it is yet to be discovered).

Whenever multiple spliced transcripts were available for a gene, a major transcript was chosen (Atngnnnnn.1) to prevent bias towards genes that contain multiple transcripts. Likewise, only introns of major transcripts were selected.

### Word Enumeration and Scoring

The first pipeline stage employs a *radix trie *data structure [48] to enumerate all subsequences (words) of a specified length in the given DNA input sequences. For each word *w*, with *o *total occurrences in *s *sequences, a word score is computed as s*ln(s/**E**_s_(w)). The expected number of sequences containing word *w*, **E**_s_(w), is computed as the product of (1) the probability for each observed word to occur anywhere in the input sequences and (2) the total length of the sequences. This model implicitly assumes a binomial model for the word distribution, i.e., that the word probabilities are independent of the positions of the words within the sequences [49,50]. The probability is computed by using a maximum-order homogeneous Markov chain model [49] where the transition probabilities are determined using the Maximum Likelihood Method [50]. (Note that under this model, the (G+C)% biasing is achieved for any order of Markov model greater than or equal to zero, since the frequencies of individual nucleotides are taken into consideration for all orders.) The order of the Markov model was chosen by using a standard chi-square test to assess the appropriateness of Markov chains of orders 0 to 6. To provide the highest precision for computation of expected values, the highest order model that passed the chi-square test was selected. Thus, an order 6 model was selected.

A p-value for each word (representing the *probability of obtaining a score at least as high as the one observed *[51]) is calculated by using a binomial word distribution to determine the probability of obtaining at least *o *repeats in the *s *input sequences that contain *w*.

### Word Clustering

The Word Clustering stage computes a cluster for each of the top scoring words (seed words) identified in the Word Scoring phase. A cluster is computed from a seed word by determining the set of words whose Hamming distance is within a user-specified threshold. A Position/Weight Matrix (PWM) is constructed for each cluster [52], and a sequence logo is created from each PWM using the TFBS module by Lenhart and Wasserman [53]. For example, the PWM for the seed word ATTTTGTA in the 3'UTRs is as follows:



The columns of the PWM correspond to nucleotide positions and the rows correspond to the nucleotides A, C, G, and T, respectively.

### Word Location Distribution

For selected words from the different segments it was determined if they were clustered at specific locations along the corresponding sequences in which they occur. In order to detect a location bias, representative of such clusters, histograms were created to show the numbers of occurrences of a specific word at each point corresponding to a positional offset from the transcription start site (TSS). For uniformity, sequence lengths were normalized to the range [1;100], to represent the number of nucleotides between the position and the TSS.

### Co-Occurrence Analysis

The Co-Occurrence Analysis considers all non-overlapping pairs of the top ranked words and computes the expected number of sequences that contain both words. Subsequently, the observed number of sequences that contain both words is determined, and an observed-to-expected ratio is computed (using a binomial word distribution) for each word pair.

### AGRIS Lookup

Previously published and curated binding site motifs which are equal to or shorter than eight base pairs were extracted from the AGRIS AtcisDB database [25], and were compared with the word lists generated for the different segments. For each motif the corresponding entries in word list were determined and the highest scoring word was identified.

### Determine Cellular Function

The MIPS Functional Catalogue Database (FunCatDB) [54], was used for determining over-represented cellular functions in each gene list containing a particular word. The workflow of the cellular function analysis, labelled as "Cellular Function" in the larger process flow (Figure 11) is as follows. For each word in the 'top 25' lists (Table 2, 3, 4, 5, 6, 7, &8) we determined the list of genes that contained the word being analyzed in the corresponding region. Then we determined the functional category of each gene by using the functional category scheme (version 2.1) retrieved from FuncatDB. The p-values for enrichment of categories were calculated by statistical tests with the hypergeometric distribution. After filtering out p-values greater than 1E-5, results were visualized by the matrix2png software package [55].

Analysis of the correlation between word location and gene expression was done as described in [39] with the following exceptions. A larger database was constructed from 1305 available raw microarray datasets (Additional file 19) present in NASC affyarrays  and the gene expression omnibus . The p-value was calculated using a chi-squared test comparing genes 2-fold induced, 2-fold suppressed, or neutral between observed (all genes harbouring the word) and expected values (based on genomic average). The Bonferroni correction was used to adjust for multiple hypothesis testing. Microarray sources included a large tissue macro-dissection [56], and the follow-up studies on stress, hormones, and pathogens [57]. We included the laser capture microdissected tissue microarray datasets [58], the gene expression profile of the Arabidopsis root [59], analysis of brassinosteroids [60], and the numerous other experiments found in the collected dataset in the above mentioned repositories. Data were normalized using global scaling of the middle 96% data points, and then noise filtered using a t-test of signal vs. background, and a t-test of signal vs. control.

## Authors' contributions

JL contributed in the development of algorithms and models, the implementation of algorithms, generation of the results and drafting of the document. JDW contributed in the development of the models and algorithms and the implementation of the approaches. KK contributed in the development, implementation and testing of models and algorithms. XL contributed in the development of the models and algorithms for co-occurrence analysis and generated the respective data. FD contributed in the development of models and algorithms, and in the implementation of the methods. MG conducted correlation analysis between word presence/location and gene expression pattern. KE contributed the idea of Hamming-distance-based clustering. SSL contributed to the statistical foundations of the scoring model. AY's contributions include extraction of data sets, functional analysis of words, and writing the manuscript. EG contributed to writing the manuscript and integrating the identified words with existing knowledge on control of gene expression. In addition to architecting the software pipeline employed in this research, LRW contributed to the design, implementation and validation of models and algorithms (especially in the areas of word searching and word scoring) and to the writing of this manuscript.

All authors read and approved the final manuscript.

## Supplementary Material

Additional file 1 **Words discovered in 3'UTRs**. Entire set of words discovered in the 3'UTRs with occurrences, expected occurrences, scores, reverse complement information and p-value. Click here for file

Additional file 2 **Words discovered in 5'UTRs**. Entire set of words discovered in the 5'UTRs with occurrences, expected occurrences, scores, reverse complement information and p-value. Click here for file

Additional file 3 **Words discovered in introns**. Entire set of words discovered in the introns with occurrences, expected occurrences, scores, reverse complement information and p-value. Click here for file

Additional file 4 **Words discovered in core promoters**. Entire set of words discovered in the core promoters [-100;+1] with occurrences, expected occurrences, scores, reverse complement information and p-value. Click here for file

Additional file 5 **Words discovered in proximal promoters**. Entire set of words discovered in the proximal promoters [-1,000;-101] with occurrences, expected occurrences, scores, reverse complement information and p-value. Click here for file

Additional file 6 **Words discovered in distal promoters**. Entire set of words discovered in the distal promoters [-3,000;-1,001] with occurrences, expected occurrences, scores, reverse complement information and p-value. Click here for file

Additional file 7 **Words discovered in entire genome**. Entire set of words discovered in the complete genome with occurrences, expected occurrences, scores, reverse complement information and p-value. Click here for file

Additional file 8 **Words missed in 3'UTRs**. Entire set of words expected to occur but not discovered in the 3'UTRs with expected occurrences. Click here for file

Additional file 9 **Words missed in 5'UTRs**. Entire set of words expected to occur but not discovered in the 5'UTRs with expected occurrences. Click here for file

Additional file 10 **Words missed in introns**. Entire set of words expected to occur but not discovered in the introns with expected occurrences. Click here for file

Additional file 11 **Words missed in core promoters**. Entire set of words expected to occur but not discovered in the core promoters with expected occurrences. Click here for file

Additional file 12 **Word based clusters**. Word-based clusters built around 2 overrepresented words of each non-coding segment of *Arabidopsis thaliana *represented by the word cluster and the sequence logo associated with said cluster. A word in a word cluster is presented through the nucleotide sequence associated with the word, the sequence count, the overall count and the SlnSES score. Click here for file

Additional file 13 **Word co-occurrences in 3'UTRs**. Entire set of co-occurring words (taken from the top 25 words) discovered in the 3'UTRs with occurrence, expected occurrences and scores. Click here for file

Additional file 14 **Word co-occurrences in 5'UTRs**. Entire set of co-occurring words (taken from the top 25 words) discovered in the 5'UTRs with occurrence, expected occurrences and scores. Click here for file

Additional file 15 **Word co-occurrences in introns**. Entire set of co-occurring words (taken from the top 25 words) discovered in the introns with occurrence, expected occurrences and scores. Click here for file

Additional file 16 **Word co-occurrences in core promoters**. Entire set of co-occurring words (taken from the top 25 words) discovered in the core promoters with occurrence, expected occurrences and scores. Click here for file

Additional file 17 **Word co-occurrences in proximal promoters**. Entire set of co-occurring words (taken from the top 25 words) discovered in the proximal promoters with occurrence, expected occurrences and scores. Click here for file

Additional file 18 **Word co-occurrences in distal promoters**. Entire set of co-occurring words (taken from the top 25 words) discovered in the distal promoters with occurrence, expected occurrences and scores. Click here for file

Additional file 19 **NASC Microarrays**. Entire set of microarray experiments available in NASC that were used for the cellular functional analysis. Click here for file
