# Supplementary material for: The word landscape of the non-coding segments of the Arabidopsis thaliana genome
Source: BMC Genomics. 2009 Oct 8;10:463. doi: 10.1186/1471-2164-10-463 (PMC2770528; doi:10.1186/1471-2164-10-463)
Supplement: Additional file 12 — Word based clusters. Word-based clusters built around 2 overrepresented words of each non-coding segment of Arabidopsis thaliana represented by the word cluster and the sequence logo associated with said cluster. A word in a word cluster is presented through the nucleotide sequence associated with the word, the sequence count, the overall count and the SlnSES score. [file 1471-2164-10-463-S12.DOC]

## Additional File 12 – Word-based clusters

Word-based clusters built around 2 overrepresented words of each non-coding segment of *Arabidopsis thaliana* represented by the word cluster and the sequence logo associated with said cluster. A word in a word cluster is presented through the nucleotide sequence associated with the word, the sequence count, the overall count and the SlnSES score.

|  | Motif 1 | Motif 2 |
| --- | --- | --- |
| 3’ UTRs | | Word | S | O | SlnSES |  | | --- | --- | --- | --- | --- | | ATTTTGTA | 732 | 752 | 165.42 | 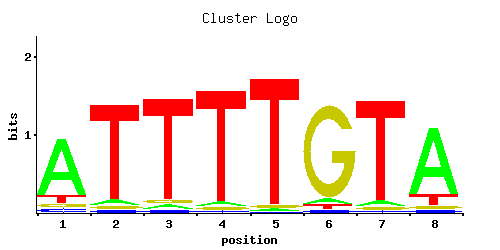 | | ATTTTTTA | 482 | 498 | 104.97 | | ATTTTGGA | 345 | 349 | 64.82 | | ATGTTGTA | 432 | 442 | 59.96 | | ATTTTCTA | 320 | 325 | 59.66 | | ATTATGTA | 372 | 379 | 47.91 | | ATATTGTA | 361 | 363 | 45.52 | | AATTTGTA | 541 | 550 | 44.56 | | ATTTTGAA | 500 | 510 | 43.37 | | AGTTTGTA | 340 | 343 | 42.22 | | ATTTTGTG | 456 | 470 | 40.67 | | ACTTTGTA | 309 | 318 | 32.66 | | ATTGTGTA | 279 | 281 | 32.54 | | CTTTTGTA | 570 | 578 | 32.37 | | ATTTTGCA | 236 | 239 | 28.21 | | ATTCTGTA | 166 | 167 | 22.55 | | ATTTCGTA | 102 | 102 | 21.49 | | ATTTTATA | 465 | 479 | 20.18 | | ATCTTGTA | 240 | 244 | 8.88 | | ATTTAGTA | 156 | 159 | 7.80 | | GTTTTGTA | 614 | 628 | 5.84 | | ATTTGGTA | 214 | 219 | 3.27 | | ATTTTGTC | 300 | 301 | -13.05 | | ATTTTGTT | 1102 | 1131 | -72.80 | | TTTTTGTA | 954 | 988 | -84.74 | | | Word | S | O | SlnSES |  | | --- | --- | --- | --- | --- | | TTCATGTT | 491 | 497 | 74.06 | 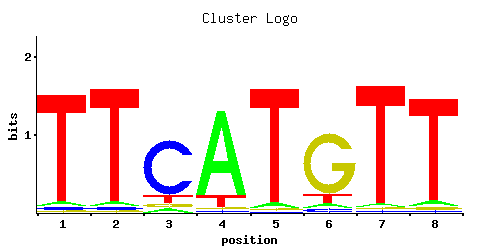 | | TTCTTGTT | 891 | 919 | 65.95 | | TTTATGTT | 832 | 862 | 53.04 | | TTCATATT | 405 | 407 | 43.53 | | TTCAAGTT | 335 | 339 | 36.58 | | TTCATTTT | 784 | 801 | 30.08 | | TTAATGTT | 418 | 424 | 27.84 | | TTGATGTT | 485 | 506 | 25.79 | | TTCATGGT | 154 | 154 | 16.81 | | TTCATCTT | 378 | 384 | 16.77 | | TTCATGCT | 114 | 114 | 13.96 | | TTCACGTT | 75 | 76 | 7.82 | | TTCCTGTT | 125 | 125 | 6.68 | | TTCGTGTT | 134 | 137 | 6.41 | | TTCAGGTT | 143 | 144 | 6.32 | | TCCATGTT | 164 | 165 | 3.74 | | TTCATGAT | 203 | 203 | 0.78 | | TGCATGTT | 103 | 103 | -5.95 | | GTCATGTT | 140 | 140 | -8.37 | | TTCATGTC | 134 | 136 | -9.23 | | CTCATGTT | 154 | 154 | -10.81 | | TTCATGTG | 192 | 197 | -13.75 | | TTCATGTA | 309 | 314 | -16.62 | | ATCATGTT | 264 | 270 | -17.87 | | TACATGTT | 210 | 216 | -32.23 | |
| 5’UTRs | | Word | S | O | SlnSES |  | | --- | --- | --- | --- | --- | | TTTCTTCA | 611 | 631 | 131.44 | 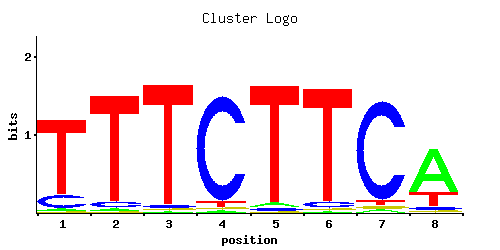 | | ATTCTTCA | 254 | 259 | 89.57 | | GTTCTTCA | 164 | 165 | 57.47 | | TTTCTCCA | 395 | 399 | 50.39 | | TTTCTTCG | 300 | 305 | 31.25 | | TTTCCTCA | 182 | 184 | 19.77 | | TGTCTTCA | 99 | 100 | 14.63 | | TTTGTTCA | 174 | 174 | 14.25 | | TTTCGTCA | 84 | 84 | 13.45 | | TTTCTGCA | 108 | 110 | 10.43 | | TTTCTTCC | 390 | 402 | 6.80 | | TTTCTACA | 94 | 96 | 4.01 | | TTACTTCA | 89 | 90 | 3.78 | | TTTCATCA | 223 | 224 | -4.49 | | TTTCTTAA | 197 | 200 | -5.30 | | TTTATTCA | 128 | 129 | -5.40 | | TTCCTTCA | 199 | 201 | -7.77 | | TTGCTTCA | 127 | 129 | -8.11 | | TATCTTCA | 140 | 142 | -11.39 | | TTTCTTGA | 204 | 213 | -18.17 | | TCTCTTCA | 401 | 406 | -20.09 | | TTTCTTTA | 278 | 287 | -23.12 | | TTTTTTCA | 306 | 312 | -33.45 | | TTTCTTCT | 1547 | 1718 | -42.46 | | CTTCTTCA | 935 | 982 | -122.82 | | | Word | S | O | SlnSES |  | | --- | --- | --- | --- | --- | | AAACCCTA | 911 | 952 | 89.78 | 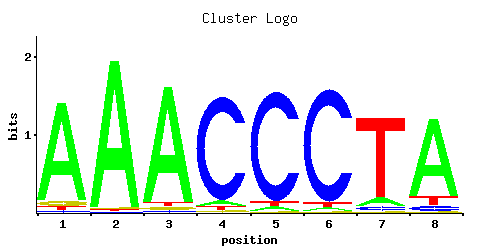 | | AATCCCTA | 127 | 128 | 62.72 | | AAAACCTA | 163 | 164 | 25.54 | | AAACCTTA | 119 | 121 | 22.99 | | AAACCATA | 94 | 95 | 15.12 | | AAATCCTA | 88 | 88 | 13.85 | | AAACCCCA | 146 | 146 | 13.62 | | AAACACTA | 98 | 99 | 10.95 | | AAGCCCTA | 80 | 80 | 10.49 | | AAACGCTA | 36 | 37 | 6.14 | | AACCCCTA | 43 | 43 | 6.10 | | GAACCCTA | 140 | 142 | 5.01 | | AAACCGTA | 26 | 27 | 4.83 | | AAACCCGA | 49 | 49 | 3.36 | | AGACCCTA | 16 | 16 | 3.27 | | ATACCCTA | 14 | 14 | 3.01 | | AAAGCCTA | 42 | 42 | 1.92 | | AAACTCTA | 98 | 99 | 1.55 | | AAACCCTG | 45 | 46 | 0.05 | | ACACCCTA | 10 | 10 | -0.22 | | AAACCCTT | 248 | 255 | -2.81 | | AAACCCAA | 256 | 261 | -6.55 | | TAACCCTA | 77 | 78 | -13.10 | | CAACCCTA | 42 | 42 | -17.96 | | AAACCCTC | 146 | 147 | -18.22 | |
| Introns | | Word | S | O | SlnSES |  | | --- | --- | --- | --- | --- | | TTTTGCAG | 3505 | 3523 | 593.06 | 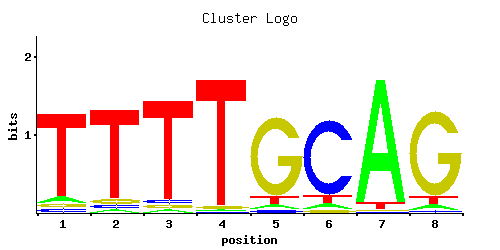 | | TGTTGCAG | 1922 | 1933 | 396.51 | | TTTTGTAG | 2556 | 2604 | 296.88 | | TTTTACAG | 1369 | 1373 | 228.39 | | TTTTTCAG | 2504 | 2534 | 223.25 | | TTTTGCTG | 1461 | 1483 | 204.74 | | TCTTGCAG | 1239 | 1243 | 192.84 | | TTATGCAG | 1026 | 1030 | 180.55 | | TATTGCAG | 1141 | 1146 | 169.75 | | TTTGGCAG | 897 | 901 | 164.22 | | TTTTCCAG | 1065 | 1075 | 163.05 | | TTGTGCAG | 1150 | 1154 | 147.98 | | TTCTGCAG | 1149 | 1157 | 135.58 | | TTTTGAAG | 1625 | 1656 | 71.84 | | ATTTGCAG | 1836 | 1850 | 66.45 | | TTTTGCGG | 228 | 230 | 42.17 | | TTTCGCAG | 322 | 323 | 32.95 | | TTTTGCCG | 173 | 174 | 24.09 | | TTTAGCAG | 316 | 320 | 14.74 | | TTTTGGAG | 859 | 871 | 6.51 | | TTTTGCAC | 445 | 447 | -28.08 | | TTTTGCAA | 1347 | 1369 | -34.61 | | CTTTGCAG | 1057 | 1060 | -56.72 | | TTTTGCAT | 1965 | 2003 | -100.21 | | GTTTGCAG | 1070 | 1072 | -122.35 | | | Word | S | O | SlnSES |  | | --- | --- | --- | --- | --- | | GTAAGTTC | 1218 | 1224 | 316.55 | 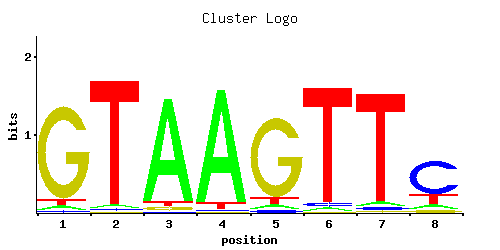 | | GTAAGTTT | 2669 | 2687 | 177.19 | | GTAAGCTC | 570 | 570 | 170.39 | | GTGAGTTC | 629 | 630 | 133.35 | | GTAAGTAC | 615 | 615 | 130.50 | | GTAAGTCC | 437 | 438 | 103.26 | | GTAATTTC | 1013 | 1023 | 99.70 | | GTATGTTC | 813 | 820 | 94.11 | | GTTAGTTC | 631 | 636 | 76.86 | | GTAAGATC | 462 | 463 | 68.47 | | GTAAATTC | 671 | 673 | 55.18 | | GTAGGTTC | 188 | 188 | 51.43 | | GTAACTTC | 509 | 511 | 49.38 | | GTAAGTGC | 302 | 302 | 48.19 | | GTACGTTC | 195 | 195 | 42.62 | | GTCAGTTC | 215 | 216 | 30.91 | | GTAAGGTC | 123 | 123 | 15.40 | | GGAAGTTC | 129 | 129 | 7.99 | | GCAAGTTC | 197 | 198 | 1.94 | | CTAAGTTC | 303 | 308 | -4.77 | | GAAAGTTC | 360 | 367 | -5.38 | | GTAAGTTA | 1064 | 1065 | -45.20 | | ATAAGTTC | 417 | 425 | -71.90 | | TTAAGTTC | 632 | 642 | -90.20 | | GTAAGTTG | 774 | 777 | -93.42 | |
| Core Promoters | | Word | S | O | SlnSES |  | | --- | --- | --- | --- | --- | | TTATATAA | 4781 | 5656 | 726.31 | 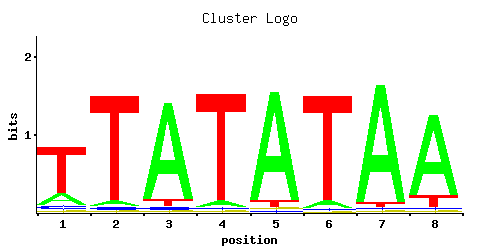 | | GTATATAA | 2471 | 2630 | 240.17 | | TTATATAC | 2480 | 2666 | 222.35 | | CTATATAA | 2332 | 2500 | 221.46 | | TTATATAG | 2349 | 2515 | 219.58 | | TTATATGA | 2766 | 2949 | 76.86 | | TTACATAA | 2410 | 2582 | 71.61 | | TTGTATAA | 2457 | 2651 | 2.50 | | TTATGTAA | 2399 | 2588 | -0.87 | | TCATATAA | 2637 | 2784 | -10.11 | | TTATACAA | 2514 | 2712 | -18.44 | | TTATCTAA | 2081 | 2214 | -24.92 | | TTAGATAA | 2045 | 2184 | -36.93 | | TGATATAA | 2443 | 2621 | -51.30 | | TTCTATAA | 2237 | 2379 | -52.76 | | TTATATCA | 2276 | 2404 | -96.80 | | TTATAGAA | 2227 | 2374 | -102.27 | | TTATATTA | 3686 | 4150 | -314.17 | | TTATAAAA | 5236 | 6357 | -315.39 | | TTTTATAA | 5199 | 6321 | -416.30 | | TTAAATAA | 4195 | 4885 | -424.94 | | TAATATAA | 3505 | 3935 | -433.15 | | TTATTTAA | 4202 | 4888 | -487.45 | | TTATATAT | 7217 | 9330 | -1645.49 | | ATATATAA | 7007 | 9027 | -1648.54 | | | Word | S | O | SlnSES |  | | --- | --- | --- | --- | --- | | CGACGTCG | 136 | 137 | 77.83 | 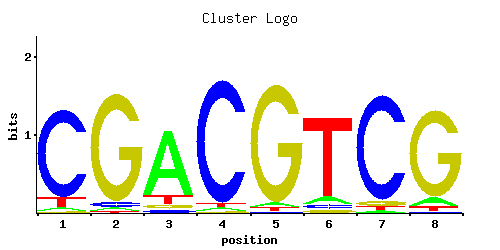 | | CGATGTCG | 44 | 44 | 43.32 | | CGACATCG | 43 | 44 | 32.40 | | CGACGCCG | 46 | 47 | 27.17 | | CGACGACG | 73 | 75 | 24.49 | | CGACGGCG | 34 | 35 | 18.12 | | CGGCGTCG | 47 | 49 | 17.39 | | CGAAGTCG | 25 | 25 | 14.99 | | CGTCGTCG | 80 | 90 | 12.45 | | CGCCGTCG | 36 | 36 | 11.86 | | CGACTTCG | 19 | 19 | 6.80 | | CTACGTCG | 12 | 12 | 5.08 | | CGACGTAG | 13 | 13 | 2.28 | | CGACGTCC | 9 | 9 | 1.68 | | CGAGGTCG | 4 | 4 | 0.60 | | CGACCTCG | 5 | 5 | 0.32 | | CGACGTGG | 25 | 25 | -1.02 | | CGACGTTG | 17 | 17 | -1.13 | | GGACGTCG | 4 | 4 | -1.35 | | CCACGTCG | 23 | 23 | -4.18 | | CAACGTCG | 11 | 12 | -4.47 | | AGACGTCG | 11 | 11 | -7.63 | | CGACGTCT | 11 | 11 | -9.23 | | CGACGTCA | 17 | 17 | -19.07 | | TGACGTCG | 22 | 22 | -21.08 | |
| Proximal Promoters | | Word | S | O | SlnSES |  | | --- | --- | --- | --- | --- | | ATTCTTCA | 1153 | 1177 | 243.52 | 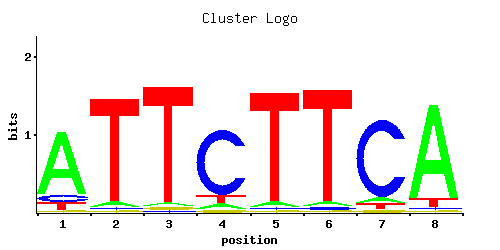 | | ATTTTTCA | 2338 | 2489 | 263.85 | | ATTCTTTA | 1316 | 1368 | 159.82 | | GTTCTTCA | 760 | 773 | 159.18 | | ACTCTTCA | 572 | 581 | 81.50 | | ATTCTACA | 757 | 777 | 81.01 | | ATTCTTGA | 938 | 963 | 80.16 | | ATTGTTCA | 859 | 880 | 78.78 | | ATACTTCA | 621 | 631 | 74.48 | | ATCCTTCA | 480 | 490 | 72.54 | | ATGCTTCA | 550 | 561 | 70.82 | | AATCTTCA | 1199 | 1230 | 70.68 | | AGTCTTCA | 506 | 514 | 63.11 | | ATTATTCA | 1479 | 1529 | 61.70 | | ATTCATCA | 1126 | 1162 | 54.47 | | ATTCTCCA | 649 | 662 | 52.47 | | ATTCCTCA | 486 | 500 | 43.50 | | TTTCTTCA | 2047 | 2162 | 38.61 | | ATTCTTAA | 1392 | 1449 | 14.34 | | ATTCGTCA | 322 | 324 | 13.14 | | ATTCTGCA | 347 | 353 | -0.27 | | ATTCTTCC | 518 | 527 | -16.74 | | ATTCTTCG | 334 | 338 | -17.14 | | ATTCTTCT | 1572 | 1642 | -169.48 | | CTTCTTCA | 1624 | 1712 | -369.78 | | | Word | S | O | SlnSES |  | | --- | --- | --- | --- | --- | | CACGTGTC | 595 | 621 | 205.35 | 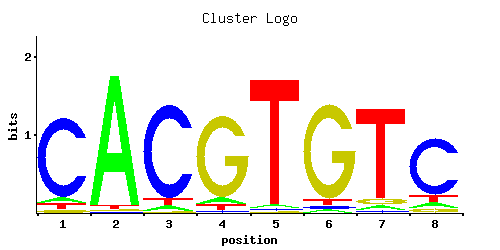 | | CACATGTC | 442 | 448 | 86.55 | | CACGTGGC | 393 | 405 | 83.14 | | CACTTGTC | 368 | 370 | 62.42 | | CATGTGTC | 330 | 335 | 46.25 | | CAAGTGTC | 279 | 280 | 44.25 | | CACGTTTC | 286 | 290 | 41.39 | | CACGTCTC | 211 | 214 | 40.22 | | CACGCGTC | 99 | 100 | 38.26 | | CACGTGAC | 251 | 255 | 35.09 | | CACGAGTC | 154 | 154 | 26.95 | | CTCGTGTC | 141 | 145 | 20.87 | | CACGTGCC | 133 | 135 | 18.57 | | CGCGTGTC | 69 | 74 | 17.76 | | CACGTATC | 155 | 158 | 16.22 | | CACCTGTC | 102 | 104 | 15.68 | | CAGGTGTC | 64 | 65 | -1.52 | | CCCGTGTC | 30 | 30 | -4.34 | | CACGGGTC | 43 | 43 | -11.60 | | GACGTGTC | 161 | 162 | -16.82 | | CACGTGTA | 350 | 355 | -37.72 | | CACGTGTG | 237 | 248 | -42.25 | | AACGTGTC | 226 | 229 | -67.01 | | TACGTGTC | 194 | 195 | -75.68 | | CACGTGTT | 347 | 358 | -101.76 | |
| Distal Promoters | | Word | S | O | SlnSES |  | | --- | --- | --- | --- | --- | | CAAGAAAC | 2910 | 3187 | 489.51 | 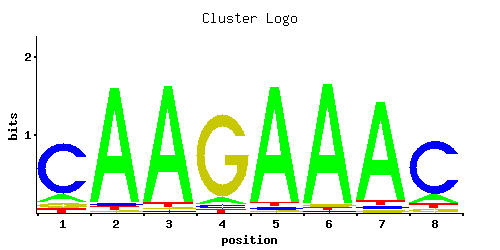 | | CAAGAAGC | 1629 | 1717 | 337.20 | | CAATAAAC | 1638 | 1700 | 318.99 | | CAAGAATC | 1993 | 2124 | 239.66 | | GAAGAAAC | 3494 | 3835 | 222.92 | | CAAGAGAC | 1180 | 1218 | 179.69 | | CTAGAAAC | 1124 | 1155 | 128.05 | | CAAGACAC | 864 | 877 | 116.41 | | CAAGATAC | 866 | 891 | 112.12 | | CGAGAAAC | 955 | 981 | 103.70 | | CAGGAAAC | 870 | 892 | 101.43 | | CATGAAAC | 1556 | 1625 | 91.21 | | CAAGCAAC | 1056 | 1094 | 84.99 | | CAAGGAAC | 945 | 965 | 81.68 | | CAACAAAC | 2206 | 2338 | 76.57 | | CAAGTAAC | 1116 | 1156 | 72.14 | | CAAGAAAT | 2735 | 2940 | 59.09 | | CACGAAAC | 634 | 647 | 36.86 | | CAAGAACC | 1480 | 1566 | 36.43 | | CCAGAAAC | 1131 | 1165 | 1.64 | | CAAGAAAG | 2577 | 2746 | -12.65 | | CAAAAAAC | 2734 | 2974 | -43.53 | | TAAGAAAC | 2568 | 2767 | -109.21 | | CAAGAAAA | 4687 | 5253 | -615.41 | | AAAGAAAC | 4243 | 4724 | -724.78 | | | Word | S | O | SlnSES |  | | --- | --- | --- | --- | --- | | CAATTTTT | 4457 | 4991 | 435.86 | 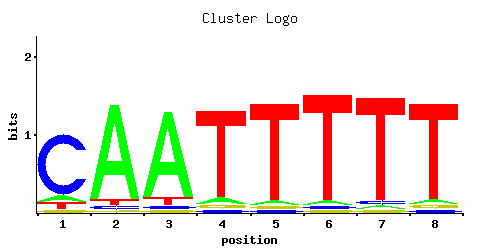 | | TAATTTTT | 6476 | 8240 | 94.11 | | CAATTTTC | 2337 | 2487 | 39.42 | | CAGTTTTT | 2526 | 2665 | 9.83 | | CAATTTCT | 2660 | 2808 | -2.91 | | CAATTGTT | 1963 | 2057 | -11.88 | | CGATTTTT | 1725 | 1800 | -26.77 | | CAATCTTT | 2511 | 2668 | -35.33 | | GAATTTTT | 3402 | 3725 | -46.05 | | CAAATTTT | 4680 | 5331 | -51.64 | | CAATTCTT | 2097 | 2215 | -54.63 | | CAATTTGT | 2023 | 2116 | -58.61 | | CCATTTTT | 3151 | 3382 | -65.64 | | CAATTTAT | 2504 | 2656 | -72.17 | | CACTTTTT | 2479 | 2654 | -90.88 | | CAACTTTT | 2797 | 3008 | -102.48 | | CAATTATT | 2597 | 2834 | -105.06 | | CAATGTTT | 2460 | 2607 | -139.87 | | CAATTTTG | 2747 | 2929 | -158.05 | | CAAGTTTT | 2829 | 3055 | -179.46 | | CTATTTTT | 3761 | 4251 | -208.86 | | CAATATTT | 3191 | 3515 | -240.19 | | CATTTTTT | 5540 | 6432 | -383.55 | | CAATTTTA | 2946 | 3150 | -432.85 | | AAATTTTT | 5291 | 6412 | -1385.80 | |
| Genome-Wide | | Word | S | O | OlnOE |  | | --- | --- | --- | --- | --- | | CCTAAACC | 5 | 5836 | 1925.48 | 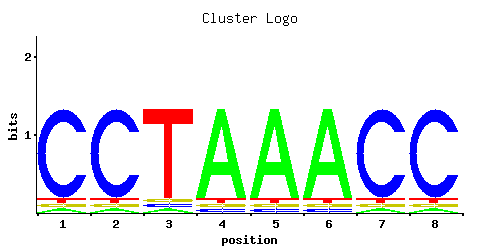 | | CTTAAACC | 5 | 4117 | 982.602 | | CCAAAACC | 5 | 6095 | 969.787 | | CCTAAATC | 5 | 3064 | 749.428 | | CCTAAAAC | 5 | 3594 | 503.783 | | CCTAATCC | 5 | 1899 | 398.032 | | CGTAAACC | 5 | 1468 | 387.395 | | CCTAACCC | 5 | 1149 | 362.675 | | CCCAAACC | 5 | 2743 | 359.335 | | CATAAACC | 5 | 3448 | 301.338 | | CCGAAACC | 5 | 1397 | 218.999 | | CCTTAACC | 5 | 1557 | 161.182 | | CCTAAGCC | 5 | 747 | 119.319 | | CCTGAACC | 5 | 1391 | 102.763 | | CCTATACC | 5 | 716 | 71.4859 | | CCTAAAGC | 5 | 1381 | 38.5076 | | CCTACACC | 5 | 849 | 23.4891 | | CCTAGACC | 5 | 588 | -14.0732 | | CCTCAACC | 5 | 1321 | -108.527 | | GCTAAACC | 5 | 1933 | -185.626 | | CCTAAACG | 5 | 997 | -205.665 | | CCTAAACT | 5 | 2809 | -474.664 | | ACTAAACC | 5 | 3857 | -485.586 | | CCTAAACA | 5 | 3142 | -787.154 | | TCTAAACC | 5 | 3642 | -826.475 | | | Word | S | O | OlnOE |  | | --- | --- | --- | --- | --- | | GGTCGAGT | 5 | 4507 | 1736.44 | 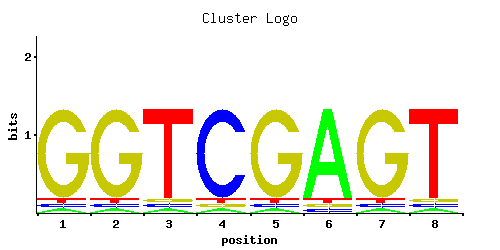 | | GATCGAGT | 5 | 2741 | 696.825 | | GGTCGGGT | 5 | 890 | 236.851 | | GGTTGAGT | 5 | 2053 | 203.404 | | GGTAGAGT | 5 | 1530 | 179.886 | | GGCCGAGT | 5 | 681 | 173.193 | | GCTCGAGT | 5 | 1067 | 123.454 | | GGTCTAGT | 5 | 1047 | 71.968 | | GGTCGAAT | 5 | 967 | 59.2328 | | GGGCGAGT | 5 | 328 | 51.5934 | | GTTCGAGT | 5 | 1240 | 46.3384 | | GGTCGATT | 5 | 913 | 46.2389 | | GGTCGCGT | 5 | 223 | 12.1502 | | GGTCCAGT | 5 | 750 | 10.8375 | | GGACGAGT | 5 | 736 | 1.34084 | | GGTCGACT | 5 | 416 | -16.8505 | | GGTCAAGT | 5 | 1609 | -43.6586 | | GGTCGTGT | 5 | 646 | -95.558 | | GGTGGAGT | 5 | 1518 | -218.611 | | GGTCGAGC | 5 | 614 | -254.824 | | AGTCGAGT | 5 | 1337 | -264.243 | | GGTCGAGG | 5 | 686 | -291.859 | | CGTCGAGT | 5 | 600 | -382.633 | | TGTCGAGT | 5 | 1021 | -480.496 | | GGTCGAGA | 5 | 966 | -561.471 | |
